# Lipidomic Profiling of Peripheral Extracellular Vesicles as a Potential Source of Biomarkers in Gaucher Disease

**DOI:** 10.3390/cells15201862

**Published:** 2026-10-13

**Authors:** Joy Guingab-Cagmat, Mariola J. Ferraro, Timothy J. Garrett, Gustavo H. B. Maegawa

**Affiliations:** 1 Department of Pathology, Immunology and Laboratory Medicine, Southeast Center for Integrated Metabolomics (SECIM), College of Medicine, University of Florida, Gainesville, FL 32610, USA; 2 Department of Microbiology and Cell Science, Institute of Food and Agricultural Sciences, University of Florida, Gainesville, FL 32610, USA; 3 Departments of Pediatrics, Vagelos College of Physicians and Surgeons, Columbia University, New York, NY 10032, USA; 4 NewYork-Presbyterian Morgan Stanley Children’s Hospital, New York, NY 10032, USA

**Keywords:** extracellular vesicles, Gaucher disease, sphingolipids, mass spectrometry, lipidomics, sphingolipids, enzyme replacement therapy

## Abstract

Extracellular vesicles (EVs) are small, membrane-enclosed nanovesicles released by cells and play an essential role in cellular communication. EVs contain condensed information about physiological/pathological states, serving as fingerprints and providing access to candidate biomarkers with potential monitoring and prognostic value. In this context, we isolated EVs from serum and urine samples from patients with Gaucher disease type 1 (GD1), a lysosomal disease (LD) caused by glucocerebrosidase deficiency. Using LC-MS/MS lipidomics, we analyzed 52 serum- and urine-derived EVs from 15 patients with GD1, including those undergoing enzyme replacement therapy (ERT). EV specimens were biologically characterized and showed increased levels of glucosylceramide and related sphingolipids. Interestingly, in the lipidomic analysis, sphingomyelin, phosphoethanolamine (PE), plasmenyl-PE, phosphatidylcholine, and some triglyceride species showed a distinct pattern in GD1. The lipidomic profiles of isolated EVs change dynamically during ERT, supporting the potential utility of EV-based biomarkers for disease staging and assessment of therapeutic response. In sum, circulating EVs with specific lipidomic profiles reflecting pathophysiological conditions are a potential source of candidate GD1 biomarkers. Given that their biogenesis is closely linked to impairment of the endosomal/lysosomal system, as classically observed in GD1 and other LDs, EV-based lipid profiling may, after validation in independent cohorts, contribute to individualized and precise monitoring of current and emerging therapies for these disorders.

## 1. Introduction

Extracellular vesicles (EVs) are nanovesicles released from cells in many different biological systems, playing a key role in cellular communication by virtue of their protein, lipid, and RNA content, which can be transferred among cells [1,2,3,4]. The molecular composition of EVs, as enclosed biomolecules, mirrors the parent cell, encoding information about the physiological or pathological state and serving as a fingerprint of the parental cell and the entire organism’s status. In addition, numerous studies have shown that EVs play a pivotal role in cellular communication, maintaining homeostasis and influencing the development and progression of pathological conditions, including infection and cancer [5,6]. Interestingly, bodily fluids are rich sources of circulating EVs, readily accessible from human blood and urine samples. The biogenesis of EVs involves an endosomal system in which early endosomes mature into late endosomes, and these vesicles subsequently generate intraluminal vesicles via endosomal membrane invagination [7,8,9,10]. Thus, the analysis of peripheral and circulating EVs may provide access to an unparalleled number of candidate biomarkers with potential diagnostic and prognostic value. Given that the pathogenic processes of lysosomal disorders (LDs) affect the endosomal/lysosomal network, EVs isolated from body fluids (plasma, serum, and urine) of affected patients conceivably carry promising biomarkers that reflect different disease stages and severities of these inborn organelle disorders.

Herein, we report the analysis of isolated peripheral EVs from body fluid specimens (serum and urine) obtained from patients with Gaucher disease (GD), an LD caused by autosomal-recessively inherited glucocerebrosidase (GCase) deficiency. GD is one of the most common LDs, characterized by a continuum of clinical findings from a perinatal lethal disorder to an asymptomatic status. GD type 1 (GD1) is the most prevalent clinical form, characterized by varying degrees and combinations of hepatosplenomegaly, anemia, thrombocytopenia, osteopenia, osteosclerosis, and an increased risk of Parkinson’s disease [11,12].

While advances in the treatment and supportive care of GD have improved, the development of specific biomarkers that predict disease complications and correlate with different disease stages remains urgently needed. GD is the first LD for which a disease-modifying therapy was developed with enzyme replacement therapy (ERT) using recombinant enzyme production [13,14]. Currently, three ERTs and two small-molecule agents are approved and available to treat affected patients [15,16]. Given the physiological relationship to the endo-lysosomal system, which is primarily affected in GD, it is conceivable that EVs reflect disturbances, including the accumulation of GCase primary substrates. In this setting, GD1 becomes an ideal model for investigating and developing EV-based biomarkers for LDs. Herein, we performed lipidomic profiling of peripheral EVs derived from serum and urine specimens of GD1 patients, aiming to identify molecular signatures with translational potential for clinical use. This approach establishes a baseline for the development of EV-based biomarkers that may be extended to other inborn lysosomal disorders.

## 2. Materials & Methods

### 2.1. Patient Demographics

The study included patients diagnosed with GD1 (*n* = 15), with varying disease onset and symptomatology. Healthy controls were used (*n* = 5; Table 1). Patients with GD1 were not significantly younger than controls (*p* = 0.169). However, a broad range of ages at diagnosis was noted (5.8 to 74.1 years; Table S1). Therefore, some degree of heterogeneity was observed in the studied cohort, a feature inherent to the type 1 clinical form of GD. Most patients presented with multisystemic problems related to GD1 (Table S1). In contrast, other patients showed few or no symptoms (Table S1). Interestingly, two subjects presented with symptoms and signs of Parkinson’s disease (Table S1). Unfortunately, given the limited clinical information, we did not feel that specific clinical correlations were possible except for the enzyme replacement therapy (ERT) treatment duration.

Control subjects had no personal history of lysosomal, hematological, hepatic, or renal disease. Specimens from controls were collected and processed using the same protocol as those from patients.

### 2.2. Specimens Analyzed

A total of 52 specimens (serum and urine) from 15 patients diagnosed with GD1 (mean age 46.5 ± 19.3 years, 6 males, 9 females) and 5 healthy controls (41.7 ± 21.1 years, 2 males, 3 females) enrolled in the lysosomal disease biobank were analyzed. Of these, 44 specimens (24 serum, 20 urine) were from GD1 patients (16 collected before and 28 during enzyme replacement therapy [ERT]), and 8 (3 serum, 5 urine) from controls. All samples were collected, aliquoted, and stored at −80 °C for an average of 10–12 years for both GD1 and control samples (Table 1). The control and GD1 samples were processed simultaneously and in the same manner. Of importance, despite the long storage time, the serum and urine aliquots from GD1 patients and controls used in the study did not undergo any freezing/thawing cycles, except for freezing for storage and thawing prior to EV isolation processing. Of the 15 GD1 subjects, 9 contributed serum, and 7 contributed urine specimens collected prior to any disease-specific treatment. The GD1 subjects who initiated enzyme replacement therapy (ERT) were clustered into 3 groups based on their time on ERT at the time of specimen collection (Table 1). The different treatment duration groups are independent, having one recruited subject contributing once to serum and urine specimens (Table 1). As intended, the lipidomics analyses of serum- and urine-derived EV specimens were performed separately. All GD1 patients received ERT, and blood and urine samples obtained on treatment were drawn immediately before an ERT infusion. The study was approved by the Columbia University IRB, which houses specimens collected under a longstanding LD natural history study of G.H.B.M. Serum samples were isolated from peripheral blood by centrifugation at 2600 rpm for 15 min at 4 °C and stored at −80 °C. Urine samples were aliquoted into cryovials and stored at −80 °C, thawed on ice immediately before analysis. The diagnosis of GD was established by demonstrating a deficiency of GCase activity in leucocytes and/or by molecular genetic testing through CAP-certified laboratories. In all GD cases, the diagnosis was confirmed by identifying pathogenic variants in *GBA1*. All pathogenic variants were previously described in ClinVar, and the HGMD entries are listed in Table S1. GD1 patients are diagnosed with GD1. The study was approved by the local ethics committee. All the participating subjects provided informed consent. Blood draw was performed at the time of clinical assessment.

### 2.3. Size-Exclusion Chromatography-Based Extracellular Vesicle (EV) Isolation

**Figure 1:**
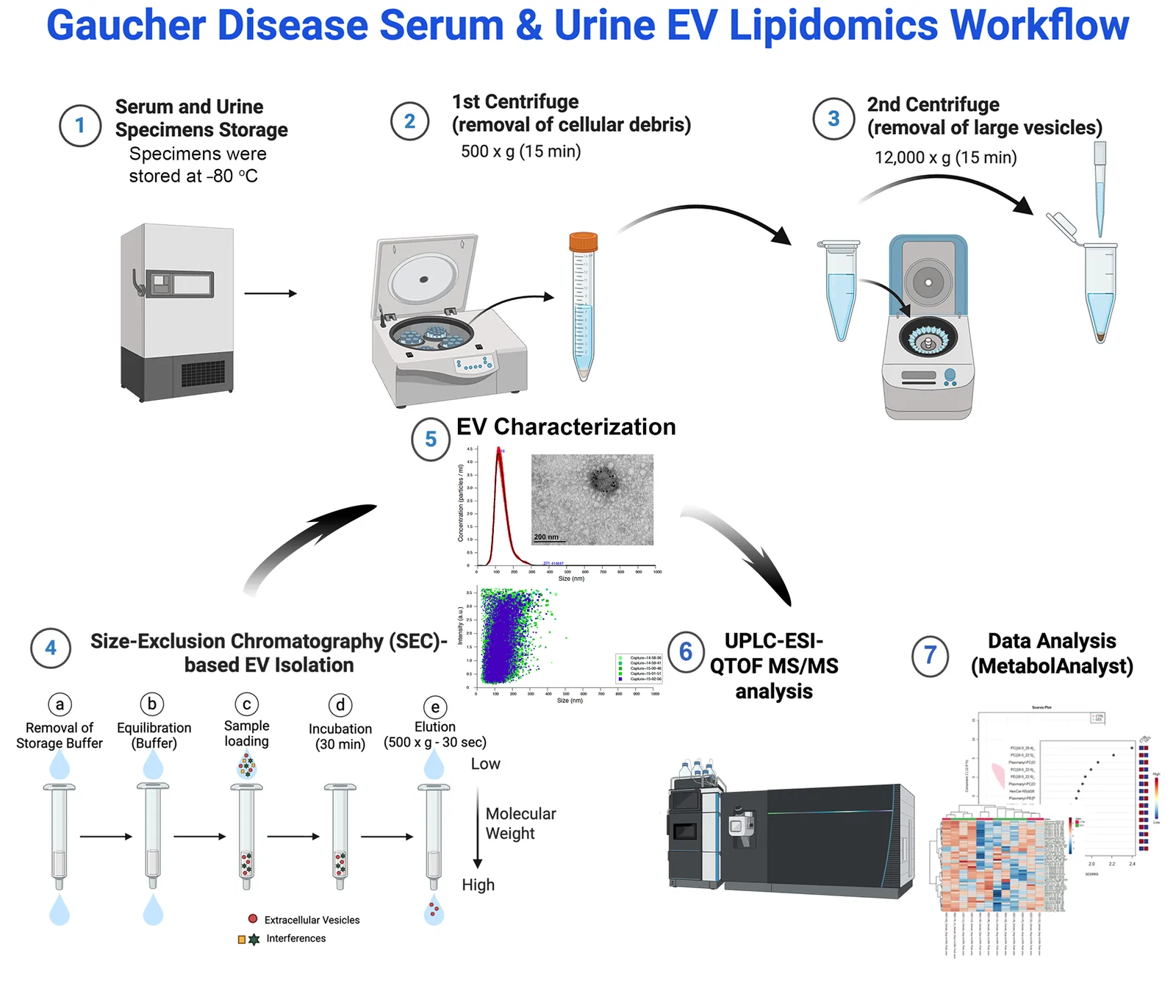
Workflow for extracellular vesicle (EV) isolation from serum and urine specimens for lipidomics analysis. (**1**) Serum and urine specimens were collected from Gaucher disease type 1 patients and controls and stored at −80 °C. (**2**) After thawing on ice, samples were processed and centrifuged (500× *g*) to remove debris. (**3**) The collected supernatant was then centrifuged to remove larger vesicles (12,000× *g*). All centrifugation steps were performed at 4 °C. (**4**) By size-exclusion chromatography (SEC) using a commercially available size-exclusion column (SmartSEC) and protocol, EVs were isolated from the serum and urine specimens. (**5**) Serum- and urine-derived EVs were then characterized. (**6**) EV lipid extracts were prepared for injection into the UPLC-qTOF MS/MS Waters instrument. (**7**) An analysis pipeline was followed using MetabolAnalyst (version 5.0).

We have followed MISEV2023 guidelines for the isolation and characterization of EVs [17]. Serum and urine specimens were thawed on wet ice, and EVs were isolated through size-exclusion chromatography (SEC) using the SmartSEC kit (System Biosciences, Palo Alto, CA, USA) according to the manufacturer’s instructions. Briefly, serum specimens were centrifuged in two stages (Figure 1). First, specimens were centrifuged at 3,000× *g* for 15 min to remove cellular debris, followed by centrifugation at 12,000× *g* for 15 min to remove large vesicles. Afterward, 250 μL aliquots of serum were added to the SEC columns. Urine samples (1000 uL aliquots) were processed using the same two-step centrifugation prior to SEC purification. EVs were eluted from the columns by centrifugation at 500× *g* for 30 s, according to the manufacturer’s protocol. The cOmplete^TM^ Mini EDTA-free Protease Inhibitor Cocktail (Roche Inc., Rockford, IL 61101 USA), containing a broad range of serine and cysteine proteases, was immediately added to the EV elution to preserve protein integrity. Isolated and intact EVs were stored at 4 °C for up to 1 week and at −20 °C for longer-term storage. For this study, lipid extraction was performed within one week of EV isolation.

### 2.4. Study Design and Workflow

First, lipidomic profiling was performed to investigate differences between EV samples isolated from serum and urine specimens obtained from subjects diagnosed with GD1 over 14 years (n = 15) and compared to healthy controls (n = 5; Table 1). Second, cross-sectional analyses were conducted to determine differences in lipidomic profiles of serum- and urine-derived EV samples obtained from GD1 patients at baseline and across three independent subcohorts grouped by enzyme replacement therapy (ERT) duration: 0–2 years, 2–5 years, and 10–14 years (Table 1). Because the ERT-duration cohorts consisted of different patients, these comparisons are cross-sectional rather than within-patient longitudinal analyses.

**Figure 2:**
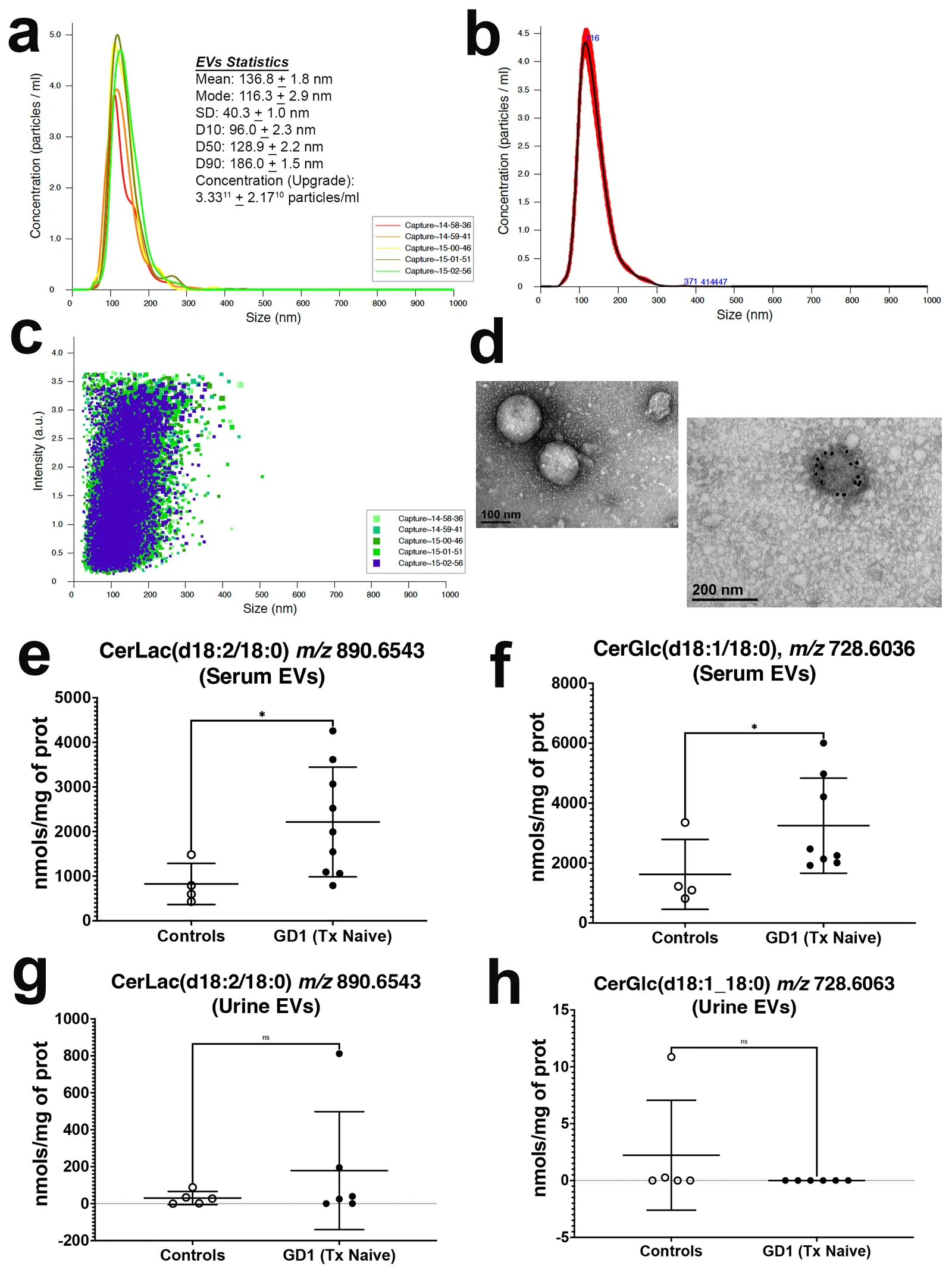
Characterization of EVs isolated from serum and urine specimens. (**a**–**c**) Nanoparticle tracking analysis (NTA) for serum- and urine-derived EVs from a Gaucher disease (GD) patient. (**a**) The EV size statistics for the 5 runs are presented as mean, mode, standard deviation, and distribution. (**b**) The averaged size distribution shows a mode of 116.3 ± 2.9 nm (mean ± SE, n = 5 captures, corresponding to approximately 3.33 ± 0.217 × 10^11^ particles per milliliter. (**c**) The scatter plot of EVs by size, with the majority of EVs being <150–200 nm. (**d**) Using transmission electron microscopy (TEM), the ultrastructural morphology of SEC-isolated EVs was evaluated. Immunogold staining was performed with anti-CD63, which binds to the EV membrane (second panel, dark dots). (**e**–**h**) Classically, due to glucocerebrosidase (GCase) deficiency, several sphingolipids accumulate, including glucosylceramide, which is the primary substrate of GCase. Significant elevations of two glucosylceramide species, such as (**e**) CerGalGlc(d18:2/18:0; 890.6543) and (**f**) CerGlc(d18:1/18:0; 728.6036), in serum-derived EVs from GD1 patients. In contrast, the same glycosylceramides CerGalGlc(d18:2/18:0; 890.6543; **g**) and CerGlc(d18:1/18:0; 728.6036; **h**), but from urine-derived EVs, showed no statistically significant differences between GD1 and age-matched healthy controls. Values are nmol/mg EV protein; bars show mean ± SD; * *p* < 0.05. *t*-test with Bonferroni correction.

Serum and urine specimens were stored in −80 °C freezers for 10–12 years prior to the analysis (Figure 1). Serum- and urine-derived EVs were isolated using the size-exclusion chromatography (SEC)-based method using a SmartSEC kit (System Biosciences, Palo Alto, CA, USA, Figure 1). Isolated EVs were characterized by using nanoparticle tracking analysis (NTA) and transmission electron microscopy (TEM) with CD63 immunogold staining (Figure 2). NTA was used to determine particle size distribution and concentration of the isolated EVs, a standard approach for EV characterization [18].

### 2.5. Protein Determination

The EV fractions from serum and urine specimens were quantified for protein using the bicinchoninic acid (BCA) reagent method with a commercially available kit (Pierce Micro BCA Protein Assay Kit, Carlsbad, CA, USA). Protein quantification was used to normalize samples for the lipidomics analysis. EV protein concentration was used (i) to express the targeted hexosylceramide measurements (Figure 2e–h) per mg of EV protein and (ii) together with NTA particle counts, to calculate particle-to-protein ratios. Protein concentration was used as the denominator for the untargeted lipidomics data, which were then normalized to the total signal for each sample.

### 2.6. Nano-Tracking Analysis (NTA)

Immediately after the isolation, EVs were examined by nano-tracking analysis (NTA). The concentration and size of EVs were determined using the NanoSight NS300 and the NanoSight 3.2 software programs (Malvern Panalytical, Malvern, UK). EVs were diluted 1:100–300 to achieve a concentration of 10–100 particles per frame in 0.1-μm filtered PBS. The camera level was set to 12, and a 488-nm laser was used.

Each sample underwent 5 repeated 1-min measurements.

### 2.7. Immunogold Labeling Negative Stain

A 400-mesh carbon-coated Formvar nickel grid was treated with a 1:10 dilution of poly-L-lysine solution (Sigma-Aldrich, St. Louis, MO, USA). The sample was then floated onto a 5 μL droplet of the sample provided. The sample was fixed to the grid using 2% paraformaldehyde in PBS and washed with PBS. The sample was incubated in a blocking solution (1% non-fat dry milk, 0.5% cold-water fish skin gelatin, 0.01% Tween-20 in PBS) and then exposed to the primary antibody against CD63 (rabbit monoclonal, SY21-02, Novus Bio., Centennial, CO, USA) at a 1:250 dilution. A negative control was established by replacing the primary antibody with PBS. Grids were washed in PBS, incubated with a 12 nm Colloidal Gold AffiniPure Goat Anti-Rabbit IgG (1:20 dilution; Jackson ImmunoResearch Laboratories, West Grove, PA, USA), washed in PBS, fixed in Trump’s fixative (Electron Microscopy Sciences, Hatfield, PA, USA), and water-washed. Once dried, the sample grid was then floated on a 10 μL droplet of 1% aqueous uranyl acetate for 30 s, stained, and removed with filter paper, air dried, and examined with a FEI Tecnai G2 Spirit Twin TEM (FEI Corp., Hillsboro, OR, USA), and digital images were acquired with a Gatan UltraScan 2k × 2k camera and Digital Micrograph software (Gatan Inc., Pleasanton, CA, USA).

### 2.8. Mass Spectrometry Metabolite Profiling

An aliquot of 100 μL of serum- and urine-derived EV underwent lipid extraction as previously reported [19]. As in previous studies [19], the chemicals used were LC-MS-grade water with 0.1% formic acid, acetonitrile (ACN), 2-propanol (IPA), and all other analytical-grade solvents, including ammonium acetate, methanol (MeOH), ammonium formate, and chloroform (CHCl_3_), purchased from ThermoFisher Scientific (Waltham, MA, USA). SPLASH^®^ LipidoMix^®^ Internal Standard Mix and all lipid injection standards were purchased from Avanti Polar Lipids, Inc. (Alabaster, AL, USA), as previously described [19].

Using the Thermo Q-Exactive Orbitrap mass spectrometer with Dionex UHPLC and autosampler, global lipidomics profiling was performed, as previously described by our group [20,21]. Aliquots from each sample were analyzed in positive and negative-heated electrospray ionization with a mass resolution of 35,000 at mass-to-charge ratio (*m*/*z*) 200 as separate injections. Separation was achieved using an ACQUITY UPLC^®^ BEH C18 column (1.7 μm × 2.1 mm × 50 mm) with a corresponding VanGuard pre-column (Waters Corporation, Milford, MA, USA) kept at 50 °C. A multi-solvent mixture of 90:8:2 IPA:ACN:water with 0.1% formic acid and 10 mM ammonium formate was used as mobile phase D, and mobile phase C was 60:40 ACN:water with 0.1% formic acid and 10 mM ammonium formate. A stepwise gradient elution was performed as follows: 0–1 min 20% D, 1–3 min 20 30% D, 3–4 min 30–45% D, 4–6 min 45–60% D, 6–8 min 60–65% D, 8–10 min 65–65% D,10–15 min 65–90% D, 15–17 min 90–98% D, 17–18 min 98–98% D, followed by 5.00 min column flush and re-equilibration. The entire runtime with equilibration was 23.00 min at a 0.5 mL/min flow rate, and the injection volumes were2 μL for positive polarity and 4 μL for negative polarity. The analyses were conducted on a Q Exactive^TM^ Orbitrap^TM^ Mass Spectrometer (Bremen(GmbH), Germany). with heated electrospray ionization coupled with a Dionex Ultimate 3000 UPLC system (Thermo Fisher Scientific, Germering, Germany) in both positive and negative ion modes using the following settings: spray voltage = 3,500 V, aux gas = 5 (+mode) and 15 (−mode), sheath gas = 30 (+mode) and 25 (−mode), capillary temperature = 300 °C, sweep gas = 1 (+mode) and 0 (−mode), and S-lens RF level = 35). All samples were kept at 8 °C in the autosampler during the analysis. Mass spectra were acquired in full scan mode with 35,000 mass resolution from 200 to 2200 *m*/*z*. Triplicate full scan injections were conducted on every sample.

### 2.9. Lipidomics Analysis

For the statistical analysis, data from the positive- and negative-ion modes were analyzed separately. All subsequent data analyses were normalized to the sum of metabolites for each sample. For the lipid identification, MetaboAnalyst 5.0 [22], and later 6.0 [23] were used, as they employ internal standards to normalize lipids in UPLC-HR MS/MS open flows, including both hydrophilic interaction liquid chromatography (HILIC) and supercritical fluid chromatography (SFC), where all lipids of a single class co-elute. Hence, semi-quantitation using appropriate correction factors to account for differences in ionization efficiencies based on carbon length and the number of unsaturations may be possible in HILIC and SFC. In contrast, in reverse-phase (RP), the use of standards for normalization should not be considered quantitative. Similarly, ion mobility has been applied to lipidomics. Since ion suppression occurs in-source before separation by ion mobility, lipid standards with varying fatty acyl constituents from analytes can still be used to account for ion-suppression effects. In addition, collision cross section obtained from ion mobility can improve confidence in identifications, and ion mobility can be used to separate isomers. However, in lipidomics, there has been limited success, as higher-resolution separation by ion-mobility is needed for lipids. Through this method, we identified several sphingolipids elevated in GD1. The serum and urine were always analyzed separately, and no quantitative comparisons of lipid abundance were made between biofluids.

### 2.10. Data Processing and Quality Control

The performance of spiked internal standards in all samples throughout the sequence was examined to curate the quality of data produced. Standards confirmed reproducibility in the analysis, showing a relative standard deviation <10%. Using RawConverter [24], the data file format conversion was performed. MZmine 2 was utilized for peak picking, chromatographic alignment, and feature identification (lipids) using our custom internal library by *m*/*z*-retention time matching [24]. For statistical analysis, non-detected species (intensity = 0) were imputed by replacing zero values with half of the minimum detected value across the dataset. A feature-by-feature signal intensity filtering algorithm was conducted to remove features with significant signal contribution from the background [22,24,25].

### 2.11. Statistical Analysis

MetaboAnalyst 5.0 was used to perform statistical analysis with the following parameters: (1) peak intensity data, (2) samples in rows unpaired, (3) missing value estimation used to replace with a small value (half of the minimum positive value in the original data, none of the features were removed in this step), (4) normalized to sum (to correct the instrumental and technical variation), (5) data transformed using log transformation, and (6) autoscaled (to allow a more direct comparison between features of greatly varying intensities). MetaboAnalyst primarily identifies and maps lipids via putative annotations based on (*m*/*z*) and optional retention time (RT) data, which aligns with MSI Level 3 (putatively characterized compound classes). First, for specimen-specific normalization, protein concentrations were determined for all lipid species. In addition, normalization procedures were implemented prior to analysis. Data were stored and organized in a table, with one specimen from a subject per row and one lipid per column. Sample-specific normalization was performed using row-wise normalization and normalization to a constant sum. The data transformation consisted of log_10_ normalization, data scaling, and autoscaling to make features more comparable (Figures S1–S4). To control for multiple testing, *p*-values were adjusted within each comparison and ionization mode using the Benjamini–Hochberg false discovery rate (FDR) procedure. Features with FDR-adjusted q < 0.05 are reported as significant and depicted in Tables S2–S5; features with unadjusted *p* < 0.05 that do not meet this threshold are reported as nominal, exploratory findings. For each feature, the fold change (ratio of group means) with 95% confidence intervals and unadjusted *p*-values are reported in Tables S4, S5, S8 and S9. Principal component analysis (PCA), an unsupervised statistical model, was employed to visualize variance and emphasize lipidomic analysis variation. Heatmaps (hierarchical clustering analysis based on Student’s *t*-test) were generated using Python 3 Seaborn to visualize metabolite and lipid intensity differences between the two samples generated from positive- and negative-ion mode data. Nanoparticle tracking results are expressed as mean ± standard error (SE). The raw data were assembled in MicrosoftExcel 365. For specific and target lipids—such as the glucosylceramide species reported when characterizing the isolated EVs—one-way ANOVA followed by the Tukey post hoc test and Bonferroni correction with *p* < 0.05 for significance was applied, using GraphPad Prism 11 (Version 11.1.0 (218)).

Following MetabolAnalyst models, univariate analysis by ANOVA was performed on all data sets to identify significant metabolites with *p*-values < 0.05. Multivariate analysis was performed using principal component analysis (PCA) and partial least squares discriminant analysis (PLS-DA) to identify features contributing most to variation between the biological samples [26,27]. Given the small group sizes, PLS-DA and variable importance in projection (VIP) scores were used only as exploratory tools to rank features by their contribution to the model. VIP scores are not a test of statistical significance; features with VIP > 1.5 were considered candidate features for further evaluation.

## 3. Results

### 3.1. Characterization of Isolated EVs

NTA of a representative serum-derived EV preparation from a GD1 patient showed a modal diameter of 116.3 ± 2.9 nm, a mean of 136.8 ± 1.8 nm, and a median (D50) of 128.9 ± 2.2 nm (mean ± SE of five captures; standard deviation of the size distribution, 40.3 ± 1.0 nm), consistent with a population dominated by vesicles smaller than 200 nm (Figure 2a–c). These findings were also consistent with TEM analysis, which confirmed the presence of small EVs with the expected nanoscale range (Figure 2d). To further validate the EV images in cryo-EM, anti-CD63 gold-labeled antibodies were prepared and showed a unique binding pattern consistent with extracellular-associated surface markers (Figure 2d). No significant differences in the EV size distribution or concentration were observed between GD1 patients, controls, and different treatment groups. The particle-to-protein concentration was included in the text (3.33^11^ ± 2.17^10^ particles/µg).

### 3.2. Characteristics of Serum and Urine EVs from Gaucher Disease Type 1

In serum- and urine-derived EV specimens, 269 lipids of different classes were detected in positive mode and 115 in negative mode. Before the comparison, data normalization was applied following MetabolAnalyst workflow (Figures S1 and S2). Among the 269 lipids detected in positive-ion mode, glycosylated ceramide species were identified and quantified in the lipid extracts from serum- and urine-derived EV fractions (Figure 2e–h). Among the glycosylated ceramide species, significantly higher levels were observed in CerGlc (d18:1_18:0; *m*/*z* 890.6543) and CerLac (d18:1_18:0; *m*/*z* 728.6036) in GD1 patient-derived EVs compared with controls (Figure 2e,f). However, the same sphingolipid species showed no significant differences in EVs harvested and isolated from urine specimens of GD1 patients (Figure 2g,h).

**Figure 3:**
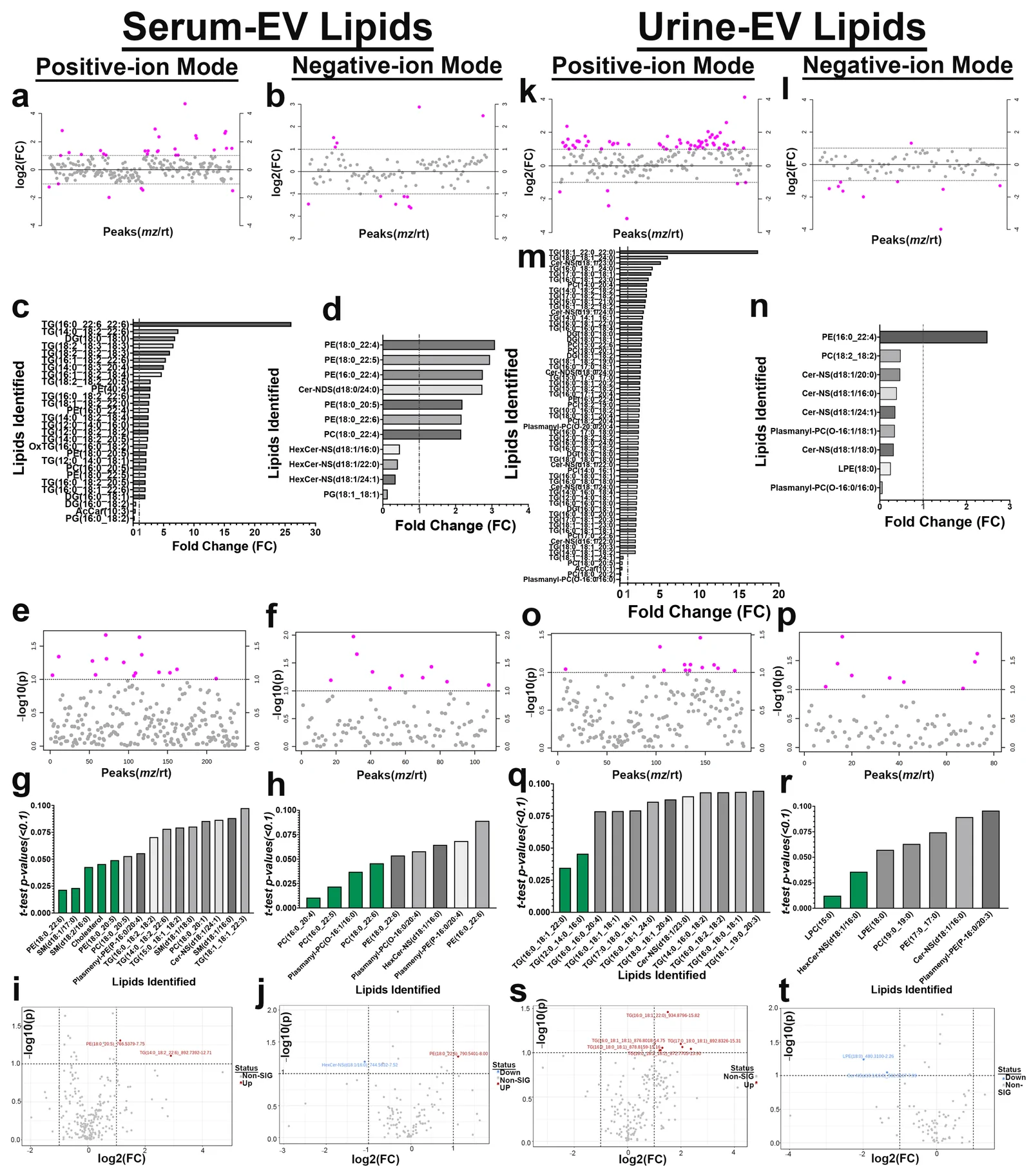
Univariate analysis of positive- and negative-ion mode detection of lipids from serum- and urine-derived extracellular vesicle (EV) specimens from patients with type 1 Gaucher disease (GD1) compared to controls. Fold-change (FC) analysis with a threshold of 2 and −2 of lipids from serum- (**a**,**b**) and urine-derived (**k**,**l**) EV specimens (pink circles, >2 or <−2) detected in positive- (**a**,**k**) and negative-ion (**b**,**l**) modes. Different lipids from serum- (**c**,**d**) and urine-derived EVs (**m**,**n**), with their respective FCs ranked from highest to lowest for positive- (**c**,**m**) and negative-ion (**d**,**n**) modes. Positive- (**e**,**o**) and negative-ion (**f**,**p**) modes detected lipids from serum- (**e**,**f**) and urine-derived EV (**o**,**p**) specimens plotted by *t*-test (−log10(*p*)). The pink circles represent *t*-tests from lipids of serum-derived EV specimens of GD1 patients that are above the threshold of 0.1. Note that the *p*-values are transformed by −log10(*p*), so that the most significant lipids (with smaller *p*-values) are plotted higher on the graph. Absolute *p*-values from *t*-test analysis are represented in histograms from the lowest to the highest for positive- (**g**,**q**) and negative-ion (**h**,**r**) modes, with green bars representing lipids from serum- (**g**,**h**) and urine-derived (**q**,**r**) EV specimens with *p* < 0.05. Positive- (**i**,**s**) and negative-ion (**j**,**t**) modes detected lipids from serum- (**i**,**j**) and urine-derived EV (**s**,**t**) specimens selected by a volcano plot by fold-change threshold (*x*, log 2(FC)) and *t*-tests threshold (*y*. −log_10_(*p*)) 0.1. The red circles represent lipids that are increased in GD1 and above the threshold (**i**,**j**,**s**). Blue circles represent lipids at decreased levels in GD1 relative to those observed in controls (**j**,**t**). The further its position is away from (0, 0), the more significant the lipid identified in the serum-derived EV specimen is.

Univariate analysis is the most common method deployed for exploratory data analysis. Univariate results are reported with adjusted *p*-values and Benjamini–Hochberg FDR-adjusted q-values of <0.05 (Tables S2–S5). Unless otherwise stated, the differences described below are nominal (unadjusted; *p* < 0.05) and should be regarded as exploratory. For the two-group analysis, which includes *t*-tests (Tables S2 and S3, Figure 3), fold-change (FC) analysis (Tables S4 and S5, Figure 3), and volcano plots (Figure 3), a combination of the first two methods was performed for the control and GD1 groups. Fold changes (FC) were calculated as the ratio of group means (unpaired design) with a threshold of 2. In positive-ion mode, among the 269 lipids identified, serum-derived EV specimens from GD1 patients showed 25 lipid species with FC > 1, and 3 with FC < 1 relative to controls, as shown in Figure 3a,c and Table S4. Using a *t*-test with an unadjusted *p*-value threshold of 0.1, 15 lipid species were identified in the serum-derived EV specimens from GD1 patients (Figure 3e,g and Table S2). In the univariate *t*-test analysis, phosphoethanolamine (PE) species PE(18:0_22:6; *m*/*z* 792.5537) and PE(18:0_20:5; *m*/*z* 766.5379) showed significant differences (*p* < 0.05) in the GD1 serum-derived EV specimens (Figure 3g, Table S2). In the same univariate *t*-test analysis, levels of sphingomyelin (SM) species SM(d18:1/17:0; *m*/*z* 717.5915) and SM(d18:2/16:0; *m*/*z* 701.5595) and cholesterol (*m*/*z* 369.3515) were nominally higher (*p* < 0.05) in GD1 serum-derived EV specimens (Figure 3e,g, Table S2). In the volcano plot, which combines the univariate FC (Figure 3a,c, Table S4) and *t*-test analysis (Figure 3e,g, Table S2), the PE species PE(18:0_20:5; *m*/*z* 766.5376) and the triglyceride (TG) species TG(14:0_18:2_22:6; *m*/*z* 892.7392) were higher in the serum-derived EV from GD1 patients (Figure 3i). In negative-ion mode, among the 115 lipids identified, the serum-derived EV specimens from GD1 patients showed 7 lipid species with FC > 1, and 4 with FC < 1 relative to controls, as shown in Figure 3b,d, and Table S4. By univariate *t*-test analysis with an unadjusted *p*-value threshold of 0.1, 9 lipid species were identified in the serum-derived EV specimens from GD1 patients (Figure 3f,h; Table S2). Phosphatidylcholine (PC) species PC(16:0_22:5; *m*/*z* 852.5763) and PC(18:0_22:6; *m*/*z* 878.5925) showed significant differences in serum-derived EV specimens from GD1 and control subjects with *p* < 0.05 (Figure 3f,h, Table S2). The levels of PC(16:0_20:4; *m*/*z* 826.5612) and plasmanyl-PC(O-16:1/16:0; *m*/*z* 762.5676) were significantly different (*p* < 0.05) in GD1 serum-derived EV specimens (Figure 3f,h, Table S2). In the volcano plot of negative-ion mode lipids from serum-derived EV specimens, PE(18:0_22:6; *m*/*z* 790.5401) was identified at higher levels, and HexCer-NS(d18:1/16:0; *m*/*z* 744.5632) at lower levels in the GD1 EVs (Figure 3j).

The univariate analysis of EVs isolated from urine specimens of GD1 patients showed a distinct profile. In positive-ion mode, among the 269 lipids identified, urine-derived EV specimens from GD1 patients showed 56 lipid species with FC > 1 and 5 with FC < 1, as shown in Figure 3k,n, and Table S5. By univariate *t*-test analysis with a threshold of 0.1, 12 lipid species were identified at different levels in urine-derived EV specimens from GD1 patients (Figure 3p,r and Table S3). TG species TG(16:0_18:1_22:0; *m*/*z* 934.8796) and TG(12:0_14:0_16:0; *m*/*z* 740.6760) showed nominal differences in the GD1 urine-derived EV specimens from GD1 patients with unadjusted with *p* < 0.05 (Figure 3p,r and Table S3). In the volcano plot, eight lipid species, TG(16:0_18:1_22:0; *m*/*z* 934.8796), TG(16:0_18:1_18:1; *m*/*z* 876.8018), TG(17:0_18:0_18:1; *m*/*z* 892.8326), TG(16:0_18:1_24:0; *m*/*z* 962.9088), TG(18:0_18:1_20:4; *m*/*z* 926.8162), Cer-NS(d18:1/23:0; *m*/*z* 636.6295), TG(16:0_18:2_18:2; *m*/*z* 872.7705), and TG(16:0_18:0_18:1; *m*/*z* 878.8159)-, showed higher levels in the urine-derived EV specimens from GD1 patients (Figure 3t). In negative-ion mode, among the 115 lipids identified, the urine-derived EV specimens from GD1 patients showed PE(16:0_22:4; *m*/*z* 766.5403) at higher levels (FC = 2.48) and 8 other lipid species at lower levels (FC < 1) than controls, as shown in Figure 3m,o and Table S5. By univariate *t*-test analysis with an unadjusted *p*-value threshold of 0.1, 7 lipid species were identified in urine-derived EV specimens from GD1 patients (Figure 3q,s and Table S3). One lysophosphatidylcholine (LPC) species, LPC(15:0; *m*/*z* 526.3153), showed nominal differences in the GD1 urine-derived EVs with *p* < 0.05 (Figure 3q,s and Table S3). The levels of HexCer-NS(d18:1/16:0; *m*/*z* 744.5632) were also nominally different (*p* < 0.05) in urine-derived EVs from GD1 patients (Figure 3q,s and Table S3). In the volcano plot of negative-ion mode lipids from urine-derived EV specimens, one lysophosphatidylethanolamine (LPE), LPE(18:0; *m*/*z* 480.3100), and one non-hydroxy ceramide (Cer-NS), Cer-NS(d18:1/16:0; *m*/*z* 582.5107), were identified at nominally lowered levels in the GD1 EVs (Figure 3u).

**Figure 4:**
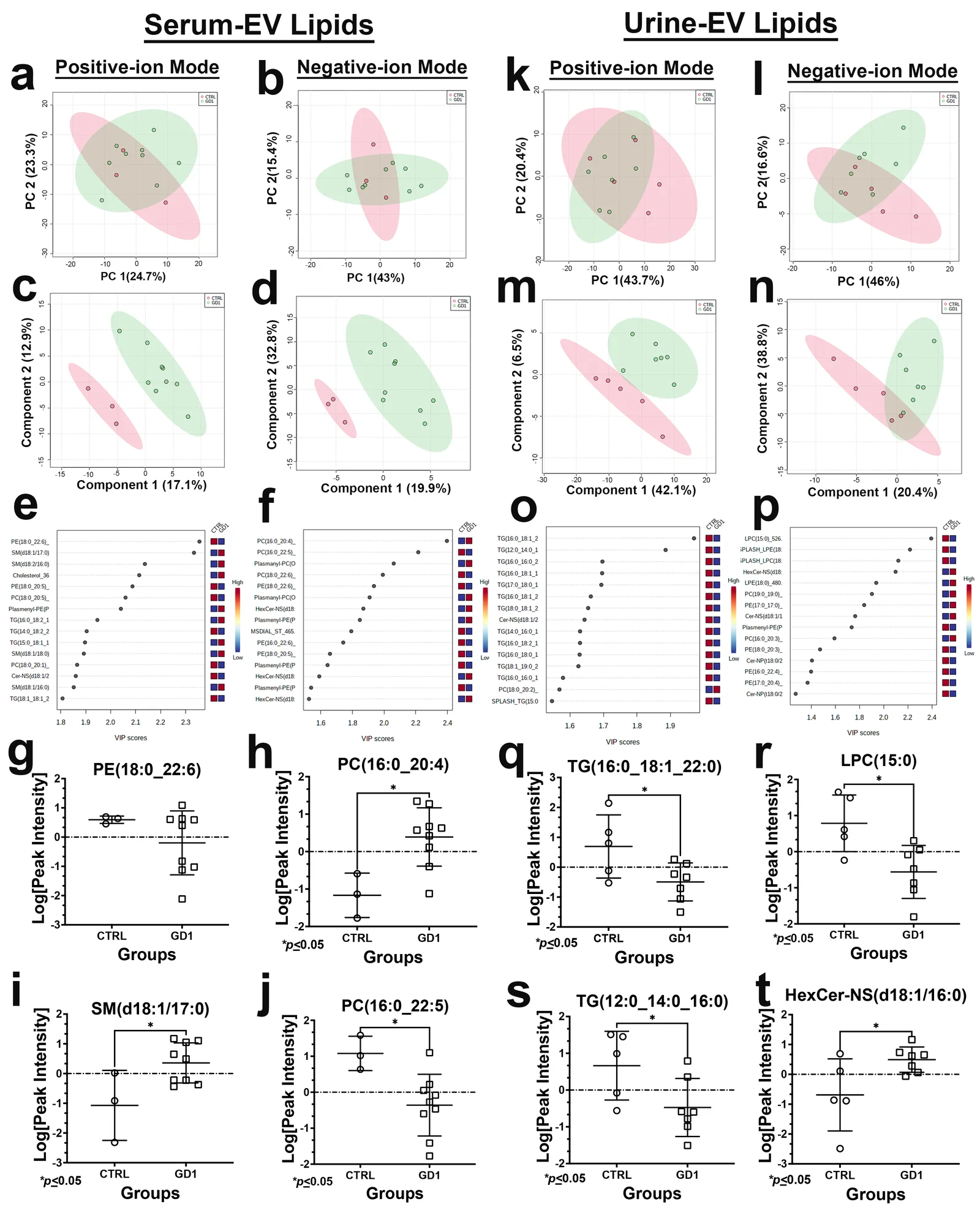
Multivariate analysis of lipids detected in serum- and urine-derived EVs from Gaucher disease type 1 (GD1) patients. Principal component analysis (PCA) plots of lipidomics profiles based on principal components 1 (PC1) and 2 (PC2) from mean intensity values of total detected lipids from GD1 subjects (green circles) and controls (red circles) of serum- (**a**,**b**) and urine-derived (**k**,**l**) EVs. Partial least squares discriminant analysis (PLS-DA) was used to explore differences between lipids extracted from serum-derived (**c**,**d**) and urine-derived (**m**,**n**) EVs. Score plots of lipids from serum-derived EVs of GD1 patients (green circles) and controls (red circles) are plotted for positive (**c**,**m**) and negative (**d**,**n**) modes. Important lipids identified by PLS-DA with a variable importance in projection (VIP) score of 1.5 or higher in both serum-derived (**e**,**f**) and urine-derived (**o**,**p**) EV specimens are shown. The colored boxes on the right of panels (**e**,**f**,**o**,**p**) indicate the relative concentrations of the corresponding lipid in each group under study. These lipids are exploratory biomarker candidates; permutation testing did not support the robustness of these models. For the serum-EV specimens, measurements of the two serum-EV lipids from GD1 and control (CTRL) groups with the highest VIP scores in positive-ion (**g**,**i**) and negative-ion (**h**,**j**) are depicted as a function of normalized Log (peak intensity). From urine-derived EVs, the highest VIP scores in positive-ion (**q**,**s**) and negative-ion (**r**,**t**) modes are also represented as a function of normalized Log (peak intensity).

Principal component analysis (PCA) was performed to assess global variation in EV lipidomic profiles from GD1 and control subjects. For the lipids extracted from serum-derived EVs, principal components 1 (PC1) and 2 (PC2) explained 48% (positive-ion mode; Figure 4a) and 58.4% (negative-ion mode; Figure 4b). For the lipid extracted from urine-derived EVs, PC1 and PC2 accounted for 64.1% (positive-ion mode; Figure 4k) and 62.6% (negative-ion mode; Figure 4l). PCA score plots of the lipidomics profile, based on PC1 and PC2, are shown in Figure 4c (positive-ion mode), d (negative-ion mode) for serum-derived EVs, using mean intensity values of the total detected metabolites. Similarly, for urine-derived EV analysis, PCA plots of the lipidomics profile based on PC1 and PC2 are depicted in Figure 4m (positive-ion mode), n (negative-ion mode), using mean intensity values for all detected metabolites.

Partial least squares discriminant analysis (PLS-DA) was conducted for both ionization modes in serum- (Figure 4e,f; Table S6) and urine-derived EVs (Figure 4o,p; Table S7). However, PLS-DA permutation testing yielded *p*-values of 1, indicating limited model robustness and suggesting that group separation should be interpreted with caution and, therefore, interpreted as only exploratory and hypothesis-generating. The apparent separation in the PLS-DA score plots therefore reflects the supervised nature of the model and does not demonstrate robust discrimination between GD1 patients and controls. In both positive- and negative-ion modes, lipids were ranked by their variable importance in projection (VIP) scores, and those with VIP > 1.5 are shown as exploratory candidate EV-isolated lipids; because the models were not supported by permutation testing, this ranking does not indicate statistical significance (Figure 4e,f for serum-derived EVs; Figure 4o,p for urine-derived EVs). In the PLS-DA model, VIP scores indicate each lipid contribution in separating the GD1 and control groups. A quantitative analysis of the two lipids from different EV specimen sets with the highest VIP scores is shown in Figure 4. Among the lipids identified from serum-derived EVs in positive-ion mode, PE(18:0_22:6; *m*/*z* 792.5537) and SM(d18:1/17:0; *m*/*z* 717.5915) are shown in Figure 4g and Figure 4i, respectively. SM(d18:1/17:0) levels were significantly higher in serum-EV specimens from GD1 patients (Figure 4i). In negative-mode, the lipid of highest VIP scores was PC(16:0_20:4; *m*/*z* 826.5612),which was significantly elevated in GD1 serum-EV specimens (Figure 4h). Interestingly, levels of another PC species, PC(16:0_22:5; *m*/*z* 852.5763), with the second highest VIP score, were significantly lower in GD1 serum-derived EV specimens (Figure 4j). The levels of remaining identified lipids from serum-derived EV specimens with VIP scores > 1.5 by PLS-DA, are shown in scattered box blots in Figures S5 (positive-ion mode) and S6 (negative-ion mode) and Table S6. Whereas among the urine-derived EVs, in positive-ion mode, the lipids with the two highest VIP scores, TG(16:0_18:1_22:0; *m*/*z* 934.8796) and TG(12:0_14:0_16:0; *m*/*z* 740.6760), showed the lowest levels in the GD1 group. In negative-ion mode, LPC(15:0; *m*/*z* 526.3153) and hex-Cer-NS(d18:1/16:0; *m*/*z* 744.5632) were significantly reduced in the GD1 specimens. The levels of remaining identified lipids from urine-EV specimens with VIP scores >1.5 by PLS-DA, in both positive- and negative-ion modes, are depicted in Figure S7 and Table S7. The features SPLASH_TG(15:0_15:0_18:1(d7)) in serum-derived EVs (Figure 4o), and iSPLASH LPE(18:1(d7); *m*/*z* 485.3381) and iSPLASH_LPC(18:1(d7); *m*/*z* 573.3907) in urine-derived EVs (Figure 4p) represent spiked deuterated internal standards and were excluded from the analysis.

**Figure 5:**
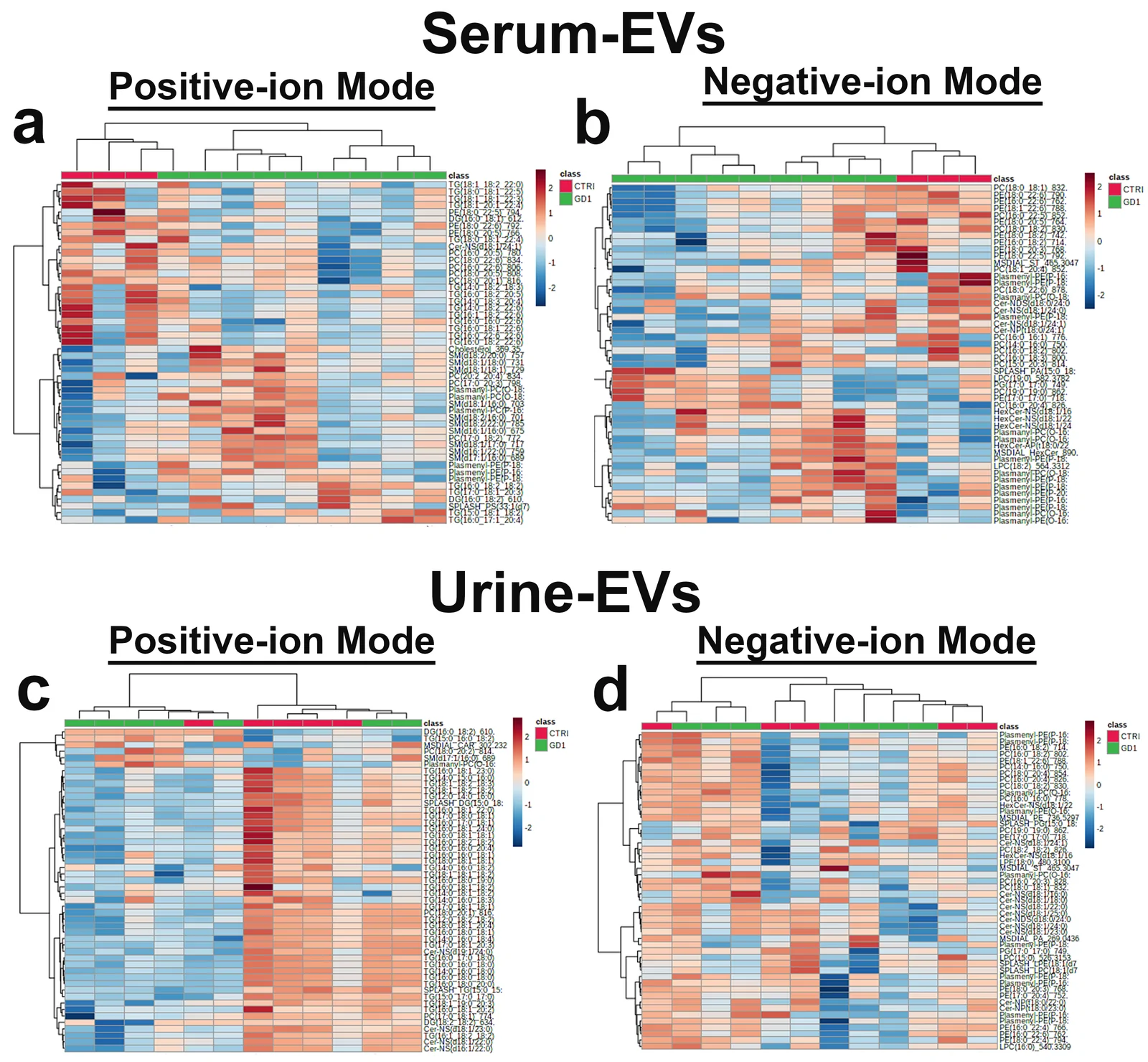
Heat maps of lipids in serum- and urine-derived EVs from Gaucher disease type 1 (GD1) patients and controls. (**a**) Heat maps of positive-ion and (**b**) negative-ion modes from lipidomics of serum-derived EVs of GD1 (green bars) and controls (red) of the top 50 features from ANOVA are shown. (**c**) Heat maps of positive-ion and (**d**) negative-ion modes from lipidomics of urine-derived EVs of GD1 (green bars) and controls (red) of the top 50 features from ANOVA are shown. Unknown features are represented by numbers representing their mass-to-charge ratio (*m*/*z*) and retention times.

Heat map analysis of the top 50 lipid features ranked by ANOVA *p*-values revealed signature patterns in both serum-derived (Figure 5a,b) and urine-derived EVs (Figure 5c,d). These patterns, clustering of certain lipids across groups, are consistent with, but do not by themselves establish, distinct lipidomic signatures in EVs from GD1 patients (Figure 5). Unidentified lipids are denoted by *m*/*z* and retention times. Among the identified lipids, several species were present abundantly in serum-derived and urine-derived EVs compared with controls (Figure 5). These are specific sphingomyelin (SM) species such as SM(d18:1/16:0; *m*/*z* 703) and SM(d18:2/16:0; *m*/*z* 701), plasmenyl-PE(P-16:0/20:4; *m*/*z* 724.5271) in positive-ion mode, and PC(16:0_20:4; *m*/*z* 826), HexCer-NS(d18:1/16:0; *m*/*z* 744.5632-7.52), and plasmanyl-PC(O-16:0/20:4; *m*/*z* 812.5818-7.78) in negative-ion mode, were increased in GD1 serum-derived EVs (Figure 5a,b). Conversely, some other lipids, PE(18:0_22:5; *m*/*z* 794), DG(16:0_18:1; *m*/*z*_612) in positive-ion mode, and LPC(19:0; *m*/*z* 582.3782), PG(17:0_17:0; *m*/*z* 749) in negative-ion mode, were decreased in GD1 serum-derived species (Figure 5a,b).

### 3.3. Lipidomic Profiles of Serum- and Urine-Derived EVs Across ERT-Duration Cohorts of Patients with Gaucher Disease Type 1

To explore ERT-associated differences, serum-derived EVs from patients with GD1 were compared cross-sectionally before ERT and across three ERT-duration cohorts: 0–2 years, 2–5 years, and 10–14 years. Details about the samples are described in Table 1. The ERT-duration cohorts comprised different GD1 patients, and the numbers of unique patients per group are shown in Table 1. Control specimens were included as a reference group (serum *n* = 3; urine *n* = 5). These treatment intervals were based on the availability of specimens from GD1 patients.

**Figure 6:**
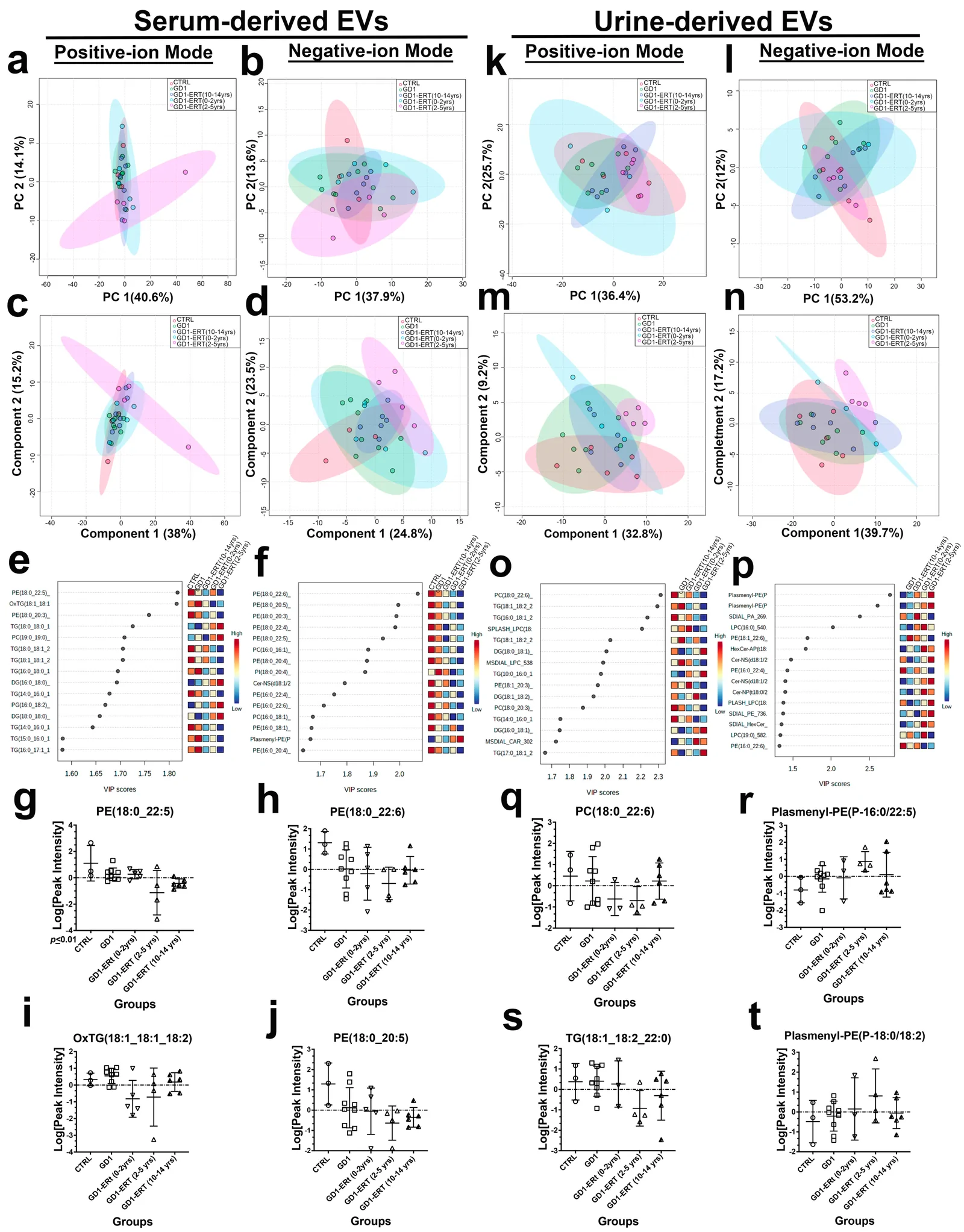
Multivariate analysis of lipids detected in serum- and urine-derived EVs from Gaucher disease type 1 (GD1) patients grouped by ERT periods. Principal component analysis (PCA) plots of lipidomics profile based on principal components 1 (PC1) and 2 (PC2) from mean intensity values of total detected lipids from controls (CTRL; red circles) and GD1 patients naive-to-treatment (green circles), GD1 patients on enzyme replacement therapy (ERT) for 0–2 years (blue circles), 2–5 yrs (pink circles) and 10–14 yrs (purple circles) for serum- (**a**,**b**) and urine-derived EVs (**k**,**l**). Partial least squares discriminant analysis (PLS-DA) was used to explore separation between the lipids in the serum- (**c**,**d**) and urine-derived EV specimens (**m**,**n**). Variable importance in projection (VIP) score plots for lipids from serum- (**e**,**f**) and urine-derived EVs (**o**,**p**) of CTRLs and GD1 patients under ERT for different time periods, as above, are plotted in the same manner for the positive and negative modes. The colored boxes on the right of panels (**e**,**f**,**o**,**p**) indicate the relative concentrations of the corresponding lipid in each group under study. Fifteen (positive) and fifteen (negative) lipids from PLS-DA had a VIP score ≥ 1.5 and are considered exploratory candidate biomarkers. Lipids from serum-EVs ((**g**,**i**) in positive-ion mode; (**h**,**j**) in negative-ion mode) and urine-EVs ((**q**,**s**) in positive-ion mode; (**r**,**t**) in negative-ion mode) of GD1 patients with the highest VIP scores were quantified in the different treatment groups and are shown as a function of normalized intensity signal (Log). For specific lipid measurements, one-way ANOVA was used to calculate significance and *p*-values.

In positive-ion mode, lipids identified from serum-derived EVs showed limited separation among treatment groups with the exception of the longest cohort, which exhibited partial clustering distinct from naive-to-treatment GD1 samples (10–14 years cohort; Figure 6a). In contrast, in negative-ion mode, lipidomics analyses revealed greater separation between the GD1-treated-naïve (baseline) and the ERT-treated cohorts across different intervals (Figure 6b). Similar clustering patterns were also observed in the urine-derived EVs in both positive-ion (Figure 6k) and negative-ion modes (Figure 6l). PCA score plots of the lipidomics profile, based on PC1 and PC2, are shown in Figure 6c (positive-ion mode) and Figure 6d (negative-ion mode), for serum-derived EVs, using mean intensity values of the total detected lipids. Similarly, for the lipidomics analysis of urine-derived EV specimens, PCA plots of the lipidomic profiles are shown in Figure 6m (positive-ion mode) and Figure 6n (negative-ion mode), using mean intensity values for all detected lipids. PLS-DA analysis of the positive- and negative-ion modes data sets from serum-derived EVs, as shown in Figure 6e,f, and in Figure 6o,p from the urine-derived EVs. PLS-DA permutation test results yielded *p* = 0.73 (positive-ion mode) and *p* = 0.03 (negative-ion mode) for two components from serum-derived EV analyses. PLS-DA permutation test results yielded *p* = 0.47 (positive-ion mode) and *p* = 0.62 (negative-ion mode) for two components from urine-derived EVs. Thus, only the serum negative-ion mode model performed better than chance (*p* = 0.03); the remaining models were not supported by permutation testing, and the associated VIP rankings are exploratory. In both positive and negative-ion modes, lipids from PLS-DA were identified based on variable importance in projection (VIP) scores of 1.5 or greater, used as an exploratory cutoff to prioritize candidate EV-isolated lipids for future validation (Figure 6e,f and Table S8 for serum-derived EVs; Figure 6o,p and Table S9 for urine-derived EVs). Measurements of two lipids from serum-derived EV specimens with the highest VIP scores in positive- (Figure 6g,i) and negative-ion modes (Figure 6h,j) are shown. The PE species—PE(18:0_22:5; *m*/*z* 794.5698), PE(18:0_22:6; *m*/*z* 790.5401), and PE(18:0_20:5; *m*/*z* 764.5245)—identified in serum-derived EVs showed lower levels in the ERT-treated cohorts than at baseline (Figure 6g,h,j). In urine-EVs, two lipids with the highest VIP scores in positive- (Figure 6q,s) and negative-ion modes (Figure 6r,t) are depicted. Plasmenyl-PE species showed higher levels in the 2–5-year cohort than at baseline (Figure 6r,t). Measurements of candidate biomarker lipids identified in serum- and urine-EVs with VIP scores > 1.5 in both positive- and negative-ion modes are shown in Figures S8–S10. In urine-derived EVs, the features SPLASH_LPC(18:1(d7); *m*/*z* 529.3996), MSDIAL_LPC (*m*/*z* 538.3867), and MSDIAL_CAR (*m*/*z* 302.2324) in positive-ion mode (Figure 6o), and MSDIAL PA (*m*/*z* 269.0436), SPLASH_LPC(18:1(d7); *m*/*z* 573.3907), MSDIAL_PE_(*m*/*z* 736.5297), MSDIAL_HexCer (*m*/*z* 890.6959) in negative-ion mode (Figure 6p) represent spiked deuterated internal standards and were excluded from the analysis.

**Figure 7:**
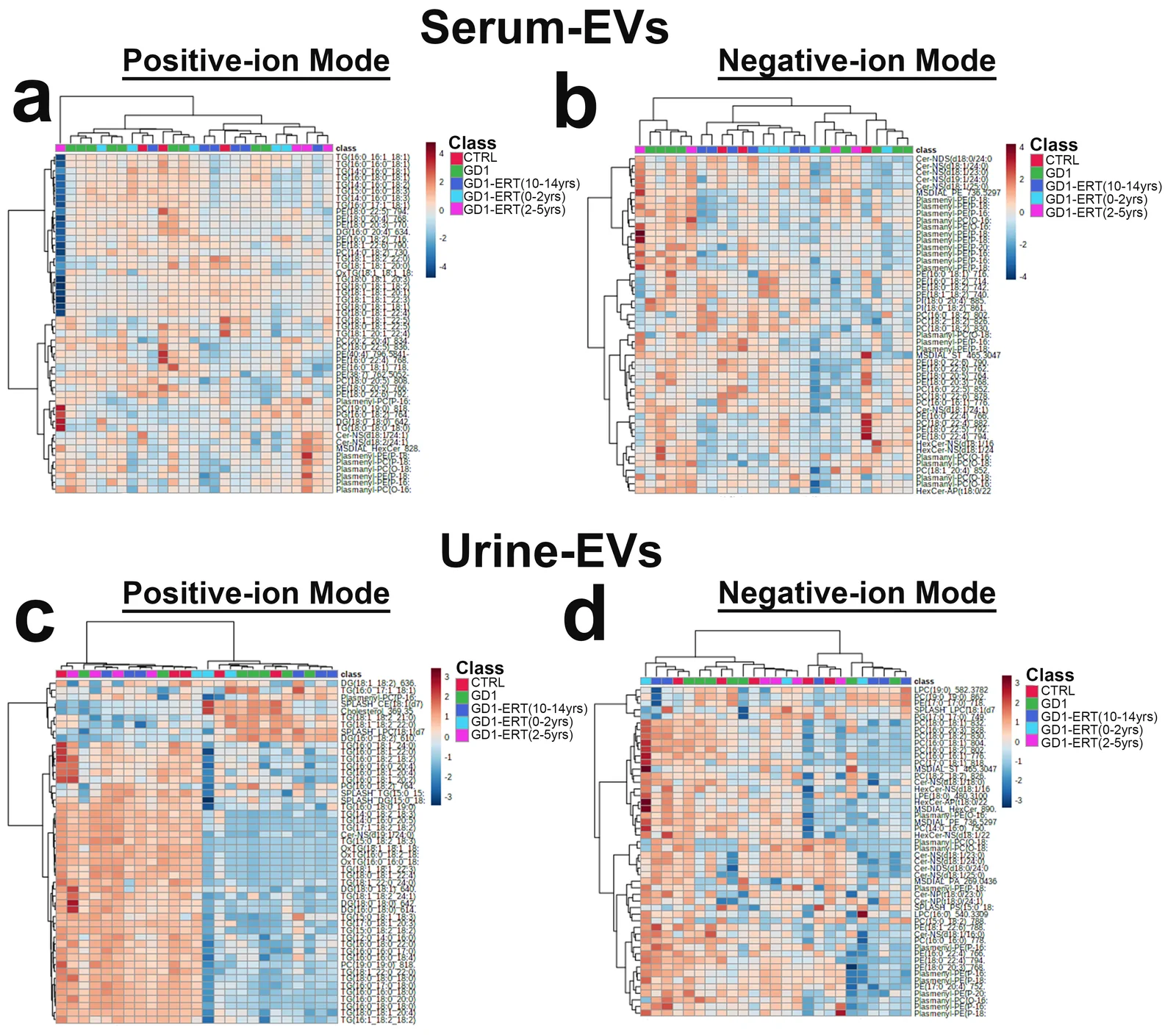
Heat maps of lipids in serum- and urine-derived EVs from controls and Gaucher disease type 1 (GD1) patients grouped by ERT duration. (**a**) Heat maps of positive-ion and (**b**) negative-ion modes from lipidomics of serum-derived EVs of controls (red), GD1 patients naive-to-treatment (green) and GD1 patients on ERT for 0–2 years (light blue), 2–5 years (magenta) and 10–14 years (dark blue) are shown. (**c**) Heat maps of positive-ion and (**d**) negative-ion modes from lipidomics of urine-derived EVs of controls (red), GD1 patients naive-to-treatment (green) and GD1 patients on ERT for 0–2 years (light blue), 2–5 years (magenta) and 10–14 years (dark blue) of the top 50 features from ANOVA are shown. Unknown features are represented by numbers representing their mass-to-charge ratio (*m*/*z*) and retention times.

Heat map analysis of the top 50 lipid features ranked by ANOVA *p*-values is shown for positive- and negative-ion modes for both positive- and negative-ion modes from both serum-derived (Figure 7a,b) and urine-derived (Figure 7c,d) EVs. These patterns suggest that ERT-associated lipidomic signatures may be detectable in circulating EV populations from different cohorts of GD1 patients. Unidentified lipid features are represented by numbers, with the first number indicating the *m*/*z* and the second indicating the retention time. Among annotated lipid species, specific sphingomyelin species such as SM(d18:1/16:0;_*m*/*z* 703.5749), and SM(d18:2/16:0; *m*/*z* 701.5595), plasmenyl-PE(P-16:0/20:4;_*m*/*z* 724.5271), in positive-ion mode (Figure 7c), and PC(16:0_20:4; *m*/*z* 826.5612), HexCer-NS(d18:1/16:0; *m*/*z* 744.5632), and plasmanyl-PC(O-16:0/20:4; *m*/*z* 812.5818), in negative-ion mode (Figure 7d), were increased in GD1 urine-derived EVs. Conversely, some other lipids, PE(18:0_22:5; *m*/*z* 794.5698) and DG(16:0_18:1; *m*/*z*_612.5564) in positive-ion mode (Figure 7a), and LPC(19:0; *m*/*z* 582.3782), and PG(17:0_17:0; *m*/*z* 749.5364) in negative-ion mode (Figure 7b), were decreased in GD1 serum-derived species analyzed.

## 4. Discussion

The study reports, to our knowledge, the first lipidomic characterization of EVs isolated from serum and urine specimens of patients with GD1, including a cross-sectional comparison of patients grouped by ERT duration. Our exploratory findings suggest that circulating and peripheral EVs can carry distinct lipid signatures associated with GD1, and that these EV lipidomic profiles differ across ERT-duration cohorts, supporting further evaluation of EVs as a minimally invasive biomarker platform for lysosomal diseases such as GD1.

Gaucher disease is an inborn lysosomal disorder caused by biallelic pathogenic variants in the *GBA1* gene, which encodes GCase, a lysosomal hydrolase that hydrolyzes glucosylceramide and glucosylsphingosine, yielding ceramide and glucose. The GCase deficiency causes progressive accumulation of glucosylceramide and its deacylated form, glucosylsphingosine, in lysosomes [11,12]. EVs contain condensed information about physiological or pathological states, serving as fingerprints of the parental cell and an entire organism. The biogenesis of EVs involves an endosomal system in which early endosomes mature into late endosomes, and these vesicles subsequently generate intraluminal vesicles via endosomal membrane invagination [7,8,9]. Consequently, the analysis of peripheral and circulating EVs provides access to an unparalleled number of biomarkers with remarkable diagnostic and prognostic value, especially in disorders affecting the lysosomal/endosomal pathways such as GD.

The availability of serum and urine specimens collected from GD1 patients before and after initiation of ERT provided a unique opportunity to evaluate EVs as a candidate biomarker for monitoring GD1 (Table 1), an area of current unmet need [28,29]. Serum and urine samples were collected and stored at −80 °C for extended periods. We are aware of the impact of specimen storage on EV concentration and physical properties [17]. Prolonged storage, even at −80 °C, and multiple freezing/thawing cycles have been shown to increase EV size, which may be due to nano- and micro-crystals in the lipid layer, resulting in particle fusion and/or membrane rupture [17]. However, −80 °C proved superior for long-term storage of samples prior to EV isolation and analysis [17]. To reduce inter-sample variability, the simultaneous processing and analysis of the serum and urine samples were performed. For the study, EVs were isolated by SEC, a method that is flexible, rapid, affordable, and compatible with clinical laboratory procedures [30,31]. For these reasons, SEC was the method of choice due to its practicality and eventual potential to adapt to a clinical laboratory setting (Figure 1). In addition, SEC can remove the most abundant proteins from a body fluid specimen while preserving vesicular structure and conformation, making it an optimal protocol for biomarker discovery and therapeutic applications [32]. Although SEC may yield lower EV recovery than some alternative isolation methods, such as ultracentrifugation, its scalability and reproducibility support its broader translational potential [31,32].

Overall, the characterization of EV number, protein content, and size showed consistent similarities between EVs from GD1 and controls (Figure 2). It is important to note that in other LDs, such as GD, differences in EV size have been reported in plasma [33]. The mean diameter of the EVs is ~136.8 ± 1.8 nm (116.3 ± 2.9 nm; mode ± SE of five NTA captures), with appropriate morphology, and EV membrane labeling for CD63 is clearly shown in TEM images (Figure 2). CD63 immunogold labeling supports the presence of tetraspanin-bearing EVs in the preparations [34,35]; however, CD63 is not specific to a single EV subtype, and a single marker is insufficient to establish EV identity or purity. We therefore use the generic term “EVs” throughout, in line with MISEV2023 [17]. For EV characterization, prior to NTA analysis, micro-BCA protein determination was performed, and samples were adjusted to a 1 µg/mL concentration. The particle-to-protein concentration was high (3.33^11^ ± 2.17^10^), generally indicating a high-purity preparation with minimal soluble protein contamination [17]. Unfortunately, due to the irreplaceable nature of the biobank specimens, representative samples were selected for EV characterization. The remaining volumes from additional specimens were insufficient for further characterization studies, including immunoblotting for additional EV-positive and -negative markers. We have therefore stated this explicitly as a limitation.

To our knowledge, this is the first analysis designed to investigate differences between affected GD1 patients and controls in serum- and urine-derived EVs (Table 1). A total of 269 lipids were identified in positive-ion LC-MS/MS mode, and the CerGlc (d18:2/18:0; *m*/*z* 890.6543) and CerLac(d18:1/18:0; *m*/*z* 728.6036) species were significantly elevated in treatment-naïve GD1 patient serum-derived EVs compared with controls (Figure 2e,f). These findings are consistent with the primary biochemical defect in GD1 and support the use of EVs to explore disease-related candidate biomarkers. Interestingly, the same sphingolipid species showed no significant differences in EVs isolated from urine specimens of GD1 patients (Figure 2g,h).

In the multivariate analysis of biomarkers for GD1, we employed the MetaboAnalyst chemometric approach, which includes two most commonly used chemometric methods—principal component analysis (PCA) and partial least squares discriminant analysis (PLS-DA) [23]. PCA is an unsupervised method that aims to find the directions of maximum variance in a data set (X) without reference to the class labels (Y) [23,26]. PLS-DA is a supervised method that uses a multiple linear regression technique to identify the direction of maximum covariance between a data set (X) and the class membership (Y). Subsequently, using the variable importance in projection (VIP) screening approach, lipids with VIP scores >1.5 were identified and ranked by their individual contribution to discriminating between the groups analyzed in the PLS-DA model [23,27]. VIP scores reflect the contribution of each lipid to the model and are not measures of statistical significance. In the serum-derived EV samples from GD1 patients, in positive-ion mode, two PE species, PE (18:0_22:6; *m*/*z* 792.5537) and PE (18:0_20:5; *m*/*z* 766.5379), showed the highest VIP scores (Figure 4g and Figure S5c; Table S6).

Interestingly, previously, PE, lyso-PE, and plasmalogen-PE were increased in GD1 and Parkinson’s disease [36]. In another study, treatment of macrophages with the GCase inhibitor conduritol-B-epoxide also increased phosphatidylcholine (PC) synthesis via PE methylation. Interestingly, the PC species PC(16:0_20:4; *m*/z 826.5612) and PC(20:2_20:4; *m*/*z* 834.5991), identified in the serum-derived EV specimens in negative- and positive-ion mode, respectively, showed increased levels in the GD1 group (Figure 4h and Figure S5d’; Table S6), whereas other PC species identified in positive-ion mode, such as PC(18:0_20:5; *m*/*z* 808.5842), PC(18:0_20:1; *m*/*z* 816.6457) and PC(18:0_22:6; *m*/*z* 834.5992), and others in negative-mode, such as PC(16:0_22:5; *m*/*z* 852.5763) and PC(18:0_18:2; *m*/*z* 830.5934), were decreased in serum-derived GD1 specimens (Figure 4j and Figures S5g,j,c’,d’ and S6b,m; Table S6). Since PC is a major structural component of biological membranes and the source of various second messengers, changes in its metabolism in macrophages may be relevant to understanding GD pathology [37]. It is possible that alterations in PC in serum-EVs may depend on the specific PC species. The plasmanyl-PC (O-16:1/16:0; *m*/*z* 762.5676) and plasmanyl-PC (O-16:0/20:4; *m*/*z* 812.5818) (Figure S6a,d; Table S6) were increased in the serum-derived EVs from GD1 patients. Previous studies have shown that reduced red blood cell and tissue plasmalogen levels—including plasmanyl-PC (ether lipids)—are observed in patients with GD1 and strongly correlate with disease severity and total body burden. The reductions in plasmalogens are associated with increased oxidative stress and altered sphingolipid metabolism (elevated glucosylceramide/ceramide ratio), both of which improve with treatment [38,39]. One hypothesis, which was not tested in the present study, is that, in the GD1 disease process, EVs contribute to the export of plasmalogens from tissue and red cells, which could account for the higher plasmanyl-PE and plasmanyl-PC levels observed here (Figure 6r,t). Experimental studies will be required to examine this hypothesis. In addition, sphingomyelin species, such as SM(d18:1/17:0; *m*/*z* 717.5915), SM(d18:2/16:0; *m*/*z* 701.5595) and others, were increased in serum-derived EV specimens from GD1 patients (Figure 4i and Figure S5a,i,l,s,u,g’; Table S6). While GD is primarily characterized by the accumulation of glucosylceramide, previous studies indicate that SM levels may decline as a consequence of increased lysosomal hydrolysis, as affected cells attempt to compensate for the buildup of other sphingolipids [40,41]. In serum-derived EV samples, HexCer-NS showed higher levels (Figure S6e,j,l). SM levels in serum-derived EVs from GD1 are uncertain and may indicate altered sphingolipid composition and membrane stability in GD1 cells [42]. In addition, one cholesterol species is increased in serum-derived EVs from patients with GD1 (Figure S5b; Table S6). GD is a lysosomal disorder that, although not a primary cholesterol disorder, significantly affects lipid metabolism and often leads to low levels of total cholesterol, HDL, and LDL. Despite low HDL (“good” cholesterol), GD patients do not appear to have an increased risk of premature cardiovascular disease.

In the urine-derived EV specimens, triglycerides TG(16:0_18:1_22:00; *m*/*z* 934.8796) and TG(12:0_14:0_16:0; *m*/*z* 740.6760) were significantly reduced in positive-ion mode (Figure 4q,s and Figure S7; Table S7). Interestingly, GD often causes dyslipidemia, including hypertriglyceridemia and low HDL cholesterol. These metabolic changes are linked to the accumulation of cellular lipids, inflammation, and the potential development of metabolic syndrome [43]. Consistent with the reduction in TG levels in serum- and urine-EVs, previous studies of GD1 bone marrow specimens showed lower triglyceride levels and markedly elevated glucocerebroside levels, as triglyceride-rich adipocytes in GD1 are progressively replaced by storage cells, leading to an overall reduction in total lipid content [44]. Consistent with the serum-derived EV species, lyso (acylated) sphingolipid species, LPC(15:0; *m*/*z* 526.3153) is nominally reduced in urine-derived EV samples (Figure 4r). Previous studies have shown that serum LPC levels are significantly decreased in GD patients compared to control groups [36]. In addition, the reduction in LPC in serum, plasma, and even CNS-derived tissues [45,46] shown in previous studies, also corresponds to the data observed in the GD1 cohort of patients here reported (Figure 4r; Table S7). Consistent with previously reported data, hexosylceramides are elevated in GD1 (Figure 4t and Table S7), as shown in previous reports [41].

Several differences were observed across ERT-duration cohorts (Figure 6); these should be interpreted in light of the cross-sectional design, small group sizes, and differences in ERT product, age, and storage time between cohorts. Using an exploratory VIP cutoff of 1.5, PE species, such as PE(18:0_22:5; *m*/*z* 794.5698), PE(18:0_20:3; *m*/*z* 770.5704) and others, showed lower levels in serum-derived EVs from ERT-treated cohorts of GD1 patients (Figure 6g,h,j, Figures S8a and S9; Table S8). The lowest levels of these two PE species were observed during the 2–5-year period cohort (Figure 6g; Table S8). An oxy-triglyceride species, OxyTG(18:1_18:1_18:2(Ke_or_Epoxy); *m*/*z* 914.7788), in serum-EVs showed a slight increase in the first years of ERT, followed by sustained reductions, albeit to a lesser extent, after the 10–14-year treatment period (Figure 6i; Table S8). In addition, a phosphatidylinositol (PI) species, PI(18:0_20:4; *m*/*z* 885.5503), was higher in serum-derived EVs from treatment-naive GD1 patients and lower in the ERT-treated cohorts (Figure S9f; Table S8).

The differences in lipid levels detected in EVs from naive-to-treatment GD1 cohorts were more evident in the shorter-duration cohorts than in the 10–14-year cohort. Previous studies have shown elevated levels of certain PI species in the serum of patients with GD1 compared with controls [36,47,48]. Earlier investigations have reported that patients receiving ERT showed a similar increase in PI species to those not receiving ERT [47]. Regarding the urine-derived EV, using the same VIP score cutoff (>1.5) in positive-ion mode, one PC species, PC(18:0_22:6; *m*/*z* 834.5992), was reduced in EVs from GD1 patients under ERT (Figure 6q; Table S9). The TG (18:1_18:2_22:0; *m*/*z* 958.8796) and several other TG species (Figure S8) also showed a decreasing pattern, consistent with that observed in other TG serum-derived EVs from GD1 (Table S8). In previous studies using plasma specimens, TG levels were not consistently elevated at baseline, and decreased after an 18-month period of ERT [43]. In negative-ion mode, two plasmalogen species, plasmenyl-PE(P-16:0/22:5; *m*/*z* 748.5299) and plasmenyl-PE(P-18:0/18:2; *m*/*z* 726.5454), showed initially elevated levels in urine-derived EVs from GD1 patients, with differing levels across ERT-duration cohorts (Figure 6r,t; Table S9). Previous studies have shown that lower levels of plasmalogens correlate negatively with increased glucosylceramide/ceramide ratio, oxidative stress, and altered sphingolipid metabolism [38,39]. Whether these differences in urine-derived EV plasmalogens reflect a response to ERT will require longitudinal, within-patient studies (Figure 6r,t).

Importantly, ERT-associated differences cannot be equated with treatment response. Therapeutic goals in GD1 include improvement or normalization of hemoglobin concentration, platelet count, and liver and spleen volumes; prevention of bone crises; and improvement in bone mineral density and quality of life. Plasma lyso-Gb1 is the most sensitive and specific established biomarker of GD1 burden and therapeutic response. Unfortunately, the lack of plasma lyso-Gb1 and clinical endpoints for EV lipids does not allow for identifying correlations regarding treatment response. Current monitoring of GD1 combines clinical and imaging assessments with plasma biomarkers, primarily lyso-Gb1, complemented by chitotriosidase (uninformative in individuals homozygous for the *CHIT1* 24-bp duplication) and CCL18. These markers mainly reflect the burden of storage macrophages and respond well to therapy, but they correlate poorly with skeletal disease (bone crises, avascular necrosis, osteopenia) and do not capture the risk of Parkinson’s disease (PD) or other neurological involvement; nor do they reliably identify patients who develop complications despite biochemical response. EV-based sampling analysis could, in principle, address part of this unmet need, because EV cargo reflects the parental cells. Urine-derived EVs can reflect the kidney and urinary tract cells, whereas cell-type-specific EV subpopulations enriched by immunocapture (e.g., from osteoclasts or neurons) might provide access to compartments that plasma lyso-Gb1 does not reflect. The data presented here represent the total EV fractions rather than cell-type-specific EVs, and the cohort is of limited size to relate lipid profiles to skeletal or neurological outcomes (i.e., only two GD1 patients presented with PD).

Practical aspects must also be considered. Compared with lyso-Gb1, a single targeted LC–MS/MS assay performed on plasma or dried blood spot samples, EV lipidomics requires additional pre-analytical centrifugation, EV SEC isolation and characterization, followed by untargeted high-resolution LC–MS with specialized data processing, thereby adding an estimated per-sample cost. Before clinical implementation, pre-analytical variables (fasting state, time of collection, processing delay, freeze–thaw cycles, and storage) would need to be standardized, reproducibility assessed across batches and laboratories, reference materials established, and the most informative candidate lipids translated into a targeted, quantitative assay.

A key question is what EV lipidomics adds to conventional lipidomics of whole serum or plasma. This concordance supports the biological plausibility of our findings [36,43,45,46,47], but it also means that, for these species, the incremental value of EV isolation has not yet been demonstrated. Because the present study did not include matched whole-serum or whole-urine lipidomics from the same specimens, nor established GD1 biomarkers such as glucosylsphingosine (lyso-Gb1) or chitotriosidase measured on the same dates, we cannot determine whether EV lipid profiles provide information beyond these measures. Potential advantages of the EV fraction are that (i) SEC depletes abundant soluble proteins and part of the lipoprotein pool, which may favor detection of lower-abundance membrane lipids; (ii) EV membranes derive from the endosomal/lysosomal and plasma-membrane compartments of the cells affected in GD1; and (iii) urine-derived EVs, which largely originate from cells of the kidney and urinary tract, sample a compartment that is not captured by circulating lipid measurements, as illustrated by the discordant hexosylceramide findings in serum- versus urine-derived EVs (Figure 2e–h). Future studies should compare matched whole-biofluid and EV lipidomics and assess whether EV lipids improve discrimination or monitoring capabilities beyond the established GD1 biomarkers.

This study has limitations. First, the cohort was small (15 GD1 patients and 5 controls, with 3–9 specimens per group), and no independent validation cohort was available; accordingly, no diagnostic performance analyses (e.g., ROC curves) were performed. Therefore, all lipids reported here should be regarded as candidate biomarkers that require validation. Second, the ERT analysis was cross-sectional: the ERT-duration cohorts comprised different patients who may differ in age, disease severity, genotype, and ERT agent, so the observed differences cannot be attributed to ERT duration itself. Third, specimens were stored at −80 °C for 10–12 years; although storage conditions were uniform, the duration of storage may affect EV integrity and lipid composition. Fourth, EV characterization was limited to NTA, TEM, and CD63 immunogold labeling of preparations. SEC-isolated EV fractions from serum can contain co-isolated lipoproteins, particularly particles that overlap with EVs in size, which may contribute TG, cholesterol, and some PC species to the measured profiles.

## 5. Conclusions

In summary, given that lysosomal disorders disrupt the endosomal/lysosomal network, EVs isolated from serum and urine specimens of GD1 patients represent a potential source of candidate biomarkers that reflect disease-associated lipid alterations and treatment-associated changes in GD1. Our exploratory findings suggest that lipidomic profiles of serum- and urine-derived EVs differ between patients grouped by ERT duration, supporting further evaluation of EV-based candidate biomarkers for monitoring therapeutic response. Similar findings have been previously reported in other sample types, including plasma and serum [36,40,41,48]. The main limitations of the study are the small cohort size, the cross-sectional design of the ERT comparison, and the absence of an independent validation cohort. In addition, further studies will be needed to confirm the reported exploratory findings from lipidomic analysis of serum- and urine-derived EV samples from prospective patients with GD1 receiving therapeutic modalities beyond ERT, including substrate reduction therapy (SRT) and upcoming therapies. Nevertheless, the present study establishes proof of principle for developing candidate EV-based lipidomic biomarkers in GD1 and demonstrates the broader potential of circulating EVs as minimally invasive tools for monitoring lysosomal disorders and treatment-associated metabolic responses.

## Figures and Tables

**Table 1:** Analysis of serum and urine EVs from Gaucher disease type 1 patients and controls.

GD1 and Control Groups				
1st analysis	GD1 × CTRLs (subject numbers)			
Specimen Type	Serum	CTRL (3)	GD1 (9)	Total Subjects = 12
	Urine	CTRL (5)	GD1 (7)	Total Subjects = 12
2nd analysis	CTRLs × GD1 Treatment Groups (subject numbers)			
Specimen Type	Serum	CTRL (3)	GD1 (15)	GD1-ERT (0–2 yrs) = 5 subjects GD1-ERT (2–5 yrs) = 4 subjects GD1-ERT (10–14 yrs) = 6 subjects
	Urine	CTRL (5)	GD1 (13)	GD1-ERT (0–2 yrs) = 3 subjects GD1-ERT (2–5 yrs) = 4 subjects GD1-ERT (10–14 yrs) = 6 subjects

Numbers of specimens are in parentheses. CTRL, control; ERT, enzyme replacement therapy; GD1, Gaucher disease type 1; yrs, years.

## Data Availability

The data that support the findings of the study here reported are available upon request from the corresponding author.

## Supplementary Materials

The following supporting information can be downloaded at: https://www.mdpi.com/article/10.3390/cells15201862/s1, **Supplementary Figure Legends:** **Figure S1**. **Box plots and kernel density plots before and after normalization of positive-ion and negative-ion mode lipid analyses of serum EV-isolated specimens from Gaucher disease type 1 (GD1) and controls.** The box plots show at most 50 features due to space limit. The density plots are based on all samples. Panel **a** depicts before and after normalizations of identified lipids in positive ion mode. Panel **b** depicts the before and after normalizations of identified lipids in negative ion mode. Selected methods: Row-wise normalization: Normalization to constant sum; Data transformation: Log_10_ Normalization; Data scaling: Autoscaling. **Figure S2**. **Box plots and kernel density plots before and after normalization of positive-ion and negative-ion mode lipid analyses of urine EV-isolated specimens from Gaucher disease type 1 (GD1) and controls.** The box plots show at most 50 features due to space limits. The density plots are based on all samples. Panel **a** depicts before and after normalizations of identified lipids in positive ion mode. Panel **b** depicts the before and after normalizations of identified lipids in negative ion mode. Selected methods: Row-wise normalization, Normalization to constant sum, Data transformation: Log_10_ Normalization, Data scaling: Autoscaling. **Figure S3**. **Box plots and kernel density plots before and after normalization of positive-ion and negative-ion mode lipid analyses of serum EV-isolated specimens from Gaucher disease type 1 (GD1) naïve and under enzyme replacement therapy and controls.** The box plots show at most 50 features due to space limit. The density plots are based on all samples. Panel **a** depicts before and after normalizations of identified lipids in negative ion mode. Panel **b** depicts the before and after normalizations of identified lipids in negative ion mode. Selected methods: Row-wise normalization: Normalization to constant sum; Data transformation: Log_10_ Normalization; Data scaling: Autoscaling. **Figure S4**. **Box plots and kernel density plots before and after normalization of positive-ion and negative-ion mode lipid analyses of urine EV-isolated specimens from Gaucher disease type 1 (GD1) naïve and under enzyme replacement therapy and controls.** The box plots show at most 50 features due to space limits. The density plots are based on all samples. Panel **a** depicts before and after normalizations of identified lipids in negative ion mode. Panel **b** depicts the before and after normalizations of identified lipids in negative ion mode. Selected methods: Row-wise normalization, Normalization to constant sum, Data transformation: Log_10_ Normalization, Data scaling: Autoscaling. **Figure S5**. **Scattered plots of lipid measurements extracted from serum-EV obtained from Gaucher disease type 1 (GD1) patients and controls in positive-ion mode.** Measurements of selected lipids detected in positive-ion mode (**a • k**’) with Variable Importance in Projection (VIP) scores > 1.5 from serum-derived EV samples collected from GD1 subjects and controls (CTRL). As text, VIP scores assess the importance of each lipid in a Partial Least Squares-Discriminant Analysis (PLS-DA) model. See Table S6 for the ranking of VIP scores, which indicate how strongly each lipid drives separation between GD1 and CTRL groups. Normalization of intensity (peak area) expressed in Log_10_. **Figure S6**. **Scattered plots of lipid measurements extracted from serum-EV obtained from Gaucher disease type 1 (GD1) patients and controls in negative-ion mode.** Measurements of selected lipids detected in negative-ion mode (**a • m**) with Variable Importance in Projection (VIP) scores > 1.5 from serum-derived EV samples collected from GD1 subjects and controls (CTRL). As text, VIP scores assess the importance of each lipid in a Partial Least Squares-Discriminant Analysis (PLS-DA) model. See Table S6 for the ranking of VIP scores, which indicate how strongly each lipid drives separation between GD1 and CTRL groups. Normalization of intensity (peak area) expressed in Log_10_. **Figure S7**. **Scattered plots of lipid measurements extracted from urine-EV obtained from Gaucher disease type 1 (GD1) patients and controls.** Measurements of selected lipids detected in positive- (**a – q**) and negative-ion (**r – z**) modes with Variable Importance in Projection (VIP) scores > 1.5 from urine-derived EV samples collected from GD1 subjects and controls (CTRL). As text, VIP scores assess the importance of each lipid in a Partial Least Squares-Discriminant Analysis (PLS-DA) model. See Table S7 for the ranking of VIP scores, which indicate how strongly each lipid drives separation between GD1 and CTRL groups. Normalization of intensity (peak area) expressed in Log_10_. **Figure S8**. **Scattered plots of lipid measurements extracted from serum-EV obtained from Gaucher disease type 1 (GD1) patients treated in different time periods in positive-ion mode.** (**a – u**) Measurements of selected lipids detected in positive-ion mode with Variable Importance in Projection (VIP) scores > 1.5 from serum-derived EV samples collected from GD1 patients before and after different time periods of enzyme replacement therapy (ERT) and controls (CTRL). As text, VIP scores assess the importance of each lipid in a Partial Least Squares-Discriminant Analysis (PLS-DA) model. See Table S8 for the ranking of VIP scores, which indicate how strongly each lipid drives separation between GD1 treatment groups and CTRL. Normalization of intensity (peak area) expressed in Log_10_. **Figure S9**. **Scattered plots of lipid measurements extracted from serum-EV obtained from Gaucher disease type 1 (GD1) patients treated in different time periods in negative-ion mode.** (**a – n**) Measurements of selected lipids detected in negative-ion mode with Variable Importance in Projection (VIP) scores > 1.5 from serum-derived EV samples collected from GD1 patients before and after different time periods of enzyme replacement therapy (ERT) and controls (CTRL). As text, VIP scores assess the importance of each lipid in a Partial Least Squares-Discriminant Analysis (PLS-DA) model. See Table S8 for the ranking of VIP scores, which indicate how strongly each lipid drives separation between GD1 treatment groups and CTRL. Normalization of intensity (peak area) expressed in Log_10_. **Figure S10**. **Scattered plots of lipid measurements extracted from urine-EV obtained from Gaucher disease type 1 (GD1) patients treated in different time periods.** Measurements of selected lipids detected in positive- (**a – s**) and negative-ion (**t – v**) modes with Variable Importance in Projection (VIP) scores > 1.5 from urine-derived EV samples collected from GD1 patients prior to and after different time periods of enzyme replacement therapy (ERT) and controls (CTRL). As text, VIP scores assess the importance of each lipid in a Partial Least Squares-Discriminant Analysis (PLS-DA) model. See Table S9 for the ranking of VIP scores, which indicate how strongly each lipid drives separation between GD1 treatment groups and CTRL. Normalization of intensity (peak area) expressed in Log_10_. **Supplementary Tables** **Table S1.** Demographics of Gaucher disease patients. **Table S2.** Analysis and identification of lipids from serum-derived EVs of GD1 subjects. **Table S3.** Analysis and identification of lipids from urine-derived EVs of GD1 subjects. **Table S4.** Fold-change analysis of serum EV-isolated lipids from GD1 patients in positive-ion and negative-ion modes. **Table S5.** Fold-change analysis of urine EV-isolated lipids in positive-ion and negative-ion modes. **Table S6.** PLS-DA VIP scores (>1.5) from serum EV-isolated lipids from GD1 patients and controls. **Table S7.** PLS-DA VIP scores (>1.5) from urine EV-isolated lipids from GD1 patients. **Table S8.** PLS-DA VIP scores (>1.5) from serum EV-isolated lipids from GD1 patients, treatment-naive and post-ERT. **Table S9.** PLS-DA VIP scores (>1.5) from urine EV-isolated lipids from GD1 patients, treatment-naive and post-ERT.
