# Supplementary material for: Lipidomic Profiling of Peripheral Extracellular Vesicles as a Potential Source of Biomarkers in Gaucher Disease
Source: Cells. 2026 Oct 13;15(20):1862. doi: 10.3390/cells15201862 (PMC13702868; doi:10.3390/cells15201862)
Supplement: Supplementary file 1 [file cells-15-01862-s001.zip › cells-4529485-supplementary.pdf]

SUPPLEMENTARY FILE

Title

Peripheral extracellular vesicles as a potential source of sounding and relevant biomarkers for Gaucher Disease

Author names

Joy Guingab-Cagmat[3]; Salma Begun[1]; Mariola J. Edelmann-Ferraro[2]; Timothy J. Garrett[3]; Gustavo H.B. Maegawa[1]

Institutional Affiliations

Departments of Pediatrics[1], Vagelos College of Physicians and Surgeons, Columbia University, New York, NY, 10032, USA; Department of Microbiology and Cell Science, Institute of Food and Agricultural Sciences[2]; Department of Pathology, Immunology and Laboratory Medicine, Southeast Center for Integrated Metabolomics (SECIM), College of Medicine[3]; University of Florida, Gainesville, FL, 32610, USA

Corresponding author

Gustavo H. B. Maegawa, MD, PhD, FACMG
Associate Professor of Pediatrics and Genetics
Columbia University Irving Medical Center
NewYork-Presbyterian Morgan Stanley Children's Hospital
Columbia University Vagelos, College of Physicians & Surgeons
630 West 168th Street, VP&S Bldg. 9-401C (office)/11-433 (Lab)
New York, NY 10032
Phone: 410 585 4955
Email: gmaegawa@icloud.com

Supplementary Figures

Figure S1

Figure S2

Figure S3

Figure S4

Figure S5

Figure S6

Figure S7

Figure S8

Figure S9

Figure S10

Supplementary Tables

Table S1

Table S2

Table S3

Table S4

Table S5

Table S6

Table S7

Table S8

Table S9

Supplementary Figure Legends

Figure S1. **Box plots and kernel density plots before and after normalization of positive-ion and negative-ion mode lipid analyses of serum EV-isolated specimens from Gaucher disease type I (GD-I) and controls.** The box plots show at most 50 features due to space limit. The density plots are based on all samples. Panel **a** depicts before and after normalizations of identified lipids in positive ion mode. Panel **b** depicts the before and after normalizations of

identified lipids in negative ion mode. Selected methods: Row-wise normalization: Normalization to constant sum; Data transformation: Log_{10} Normalization; Data scaling: Autoscaling.

Figure S2. **Box plots and kernel density plots before and after normalization of positive-ion and negative-ion mode lipid analyses of urine EV-isolated specimens from Gaucher disease type I (GD-I) and controls.** The box plots show at most 50 features due to space limits. The density plots are based on all samples. Panel **a** depicts before and after normalizations of identified lipids in positive ion mode. Panel **b** depicts the before and after normalizations of identified lipids in negative ion mode. Selected methods: Row-wise normalization, Normalization to constant sum, Data transformation: Log_{10} Normalization, Data scaling: Autoscaling.

Figure S3. **Box plots and kernel density plots before and after normalization of positive-ion and negative-ion mode lipid analyses of serum EV-isolated specimens from Gaucher disease type I (GD-I) naïve and under enzyme replacement therapy and controls.** The box plots show at most 50 features due to space limit. The density plots are based on all samples. Panel **a** depicts before and after normalizations of identified lipids in negative ion mode. Panel **b** depicts the before and after normalizations of identified lipids in negative ion mode. Selected methods: Row-wise normalization: Normalization to constant sum; Data transformation: Log_{10} Normalization; Data scaling: Autoscaling.

Figure S4. **Box plots and kernel density plots before and after normalization of positive-ion and negative-ion mode lipid analyses of urine EV-isolated specimens from Gaucher disease type I (GD-I) naïve and under enzyme replacement therapy and controls.** The box plots show at most 50 features due to space limits. The density plots are based on all samples. Panel **a** depicts before and after normalizations of identified lipids in negative ion mode. Panel **b** depicts the before and after normalizations of identified lipids in negative ion mode. Selected

methods: Row-wise normalization, Normalization to constant sum, Data transformation: Log_{10} Normalization, Data scaling: Autoscaling.

Figure S5. **Scattered plots of lipid measurements extracted from serum-EV obtained from Gaucher disease type 1 (GD1) patients and controls in positive-ion mode.** Measurements of selected lipids detected in positive-ion mode (**a - k**') with Variable Importance in Projection (VIP) scores > 1.5 from serum-derived EV samples collected from GD1 subjects and controls (CTRL). As text, VIP scores assess the importance of each lipid in a Partial Least Squares-Discriminant Analysis (PLS-DA) model. See Table S6 for the ranking of VIP scores, which indicate how strongly each lipid drives separation between GD1 and CTRL groups. Normalization of intensity (peak area) expressed in Log_{10}.

Figure S6. **Scattered plots of lipid measurements extracted from serum-EV obtained from Gaucher disease type 1 (GD1) patients and controls in negative-ion mode.** Measurements of selected lipids detected in negative-ion mode (**a - m**) with Variable Importance in Projection (VIP) scores > 1.5 from serum-derived EV samples collected from GD1 subjects and controls (CTRL). As text, VIP scores assess the importance of each lipid in a Partial Least Squares-Discriminant Analysis (PLS-DA) model. See Table S6 for the ranking of VIP scores, which indicate how strongly each lipid drives separation between GD1 and CTRL groups. Normalization of intensity (peak area) expressed in Log_{10}.

Figure S7. **Scattered plots of lipid measurements extracted from urine-EV obtained from Gaucher disease type 1 (GD1) patients and controls.** Measurements of selected lipids detected in positive- (**a – q**) and negative-ion (**r – z**) modes with Variable Importance in Projection (VIP) scores > 1.5 from urine-derived EV samples collected from GD1 subjects and controls (CTRL). As text, VIP scores assess the importance of each lipid in a Partial Least Squares-Discriminant Analysis (PLS-DA) model. See Table S7 for the ranking of VIP scores,

which indicate how strongly each lipid drives separation between GD1 and CTRL groups. Normalization of intensity (peak area) expressed in Log_{10}.

Figure S8. **Scattered plots of lipid measurements extracted from serum-EV obtained from Gaucher disease type 1 (GD1) patients treated in different time periods in positive-ion mode.** (**a – u**) Measurements of selected lipids detected in positive-ion mode with Variable Importance in Projection (VIP) scores > 1.5 from serum-derived EV samples collected from GD1 patients before and after different time periods of enzyme replacement therapy (ERT) and controls (CTRL). As text, VIP scores assess the importance of each lipid in a Partial Least Squares-Discriminant Analysis (PLS-DA) model. See Table S8 for the ranking of VIP scores, which indicate how strongly each lipid drives separation between GD1 treatment groups and CTRL. Normalization of intensity (peak area) expressed in Log_{10}.

Figure S9. **Scattered plots of lipid measurements extracted from serum-EV obtained from Gaucher disease type 1 (GD1) patients treated in different time periods in negative-ion mode.** (**a – n**) Measurements of selected lipids detected in negative-ion mode with Variable Importance in Projection (VIP) scores > 1.5 from serum-derived EV samples collected from GD1 patients before and after different time periods of enzyme replacement therapy (ERT) and controls (CTRL). As text, VIP scores assess the importance of each lipid in a Partial Least Squares-Discriminant Analysis (PLS-DA) model. See Table S8 for the ranking of VIP scores, which indicate how strongly each lipid drives separation between GD1 treatment groups and CTRL. Normalization of intensity (peak area) expressed in Log_{10}.

Figure S10. **Scattered plots of lipid measurements extracted from urine-EV obtained from Gaucher disease type 1 (GD1) patients treated in different time periods.** Measurements of selected lipids detected in positive- (**a – s**) and negative-ion (**t – v**) modes with Variable Importance in Projection (VIP) scores > 1.5 from urine-derived EV samples collected from GD1 patients prior to and after different time periods of enzyme replacement therapy (ERT) and

controls (CTRL). As text, VIP scores assess the importance of each lipid in a Partial Least Squares-Discriminant Analysis (PLS-DA) model. See Table S9 for the ranking of VIP scores, which indicate how strongly each lipid drives separation between GD1 treatment groups and CTRL. Normalization of intensity (peak area) expressed in Log_{10}.

Figure S1

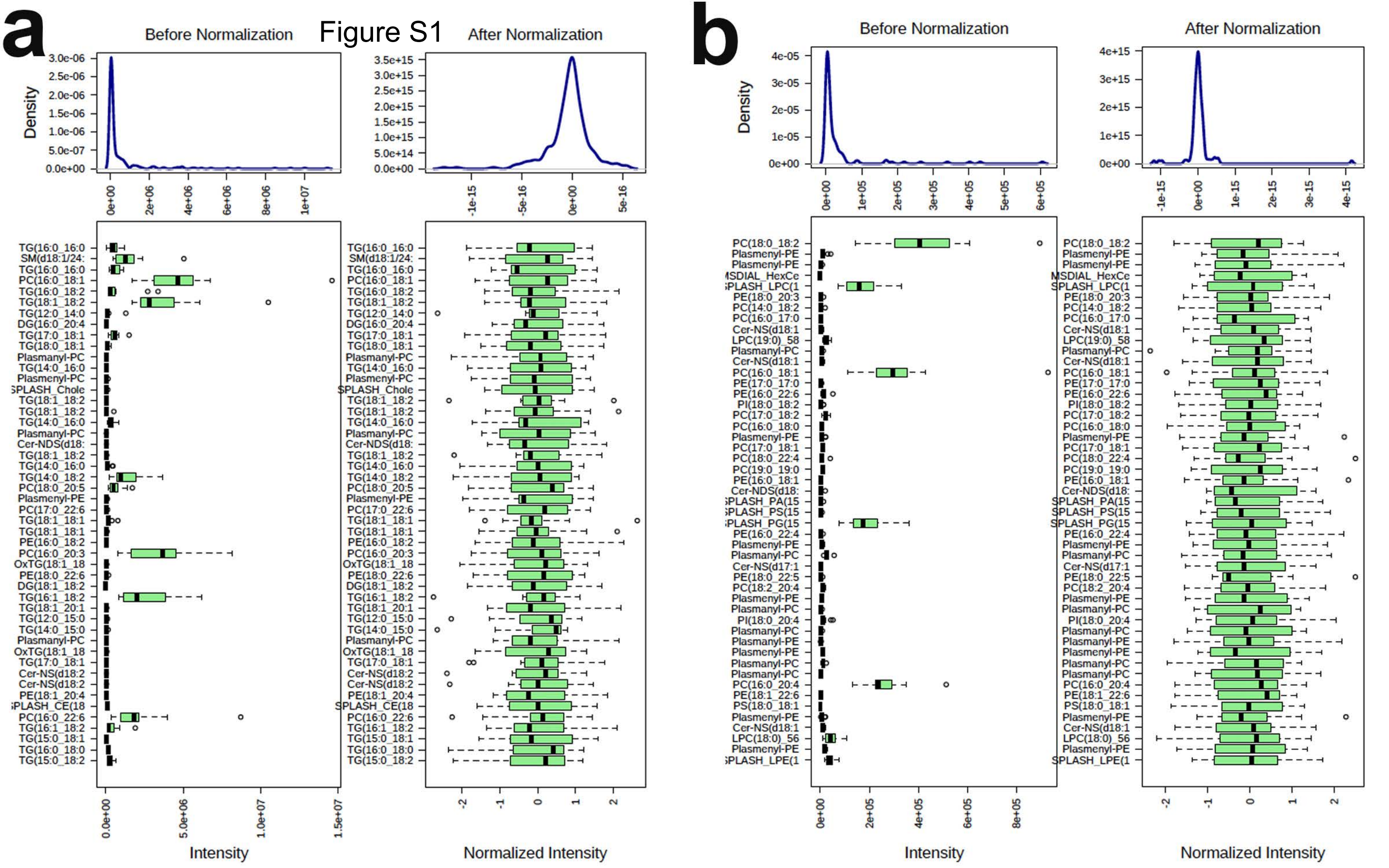
a
Before Normalization
After Normalization
Density
Intensity
Normalized Intensity
b
Before Normalization
After Normalization
Density
Intensity
Normalized Intensity

Figure S2

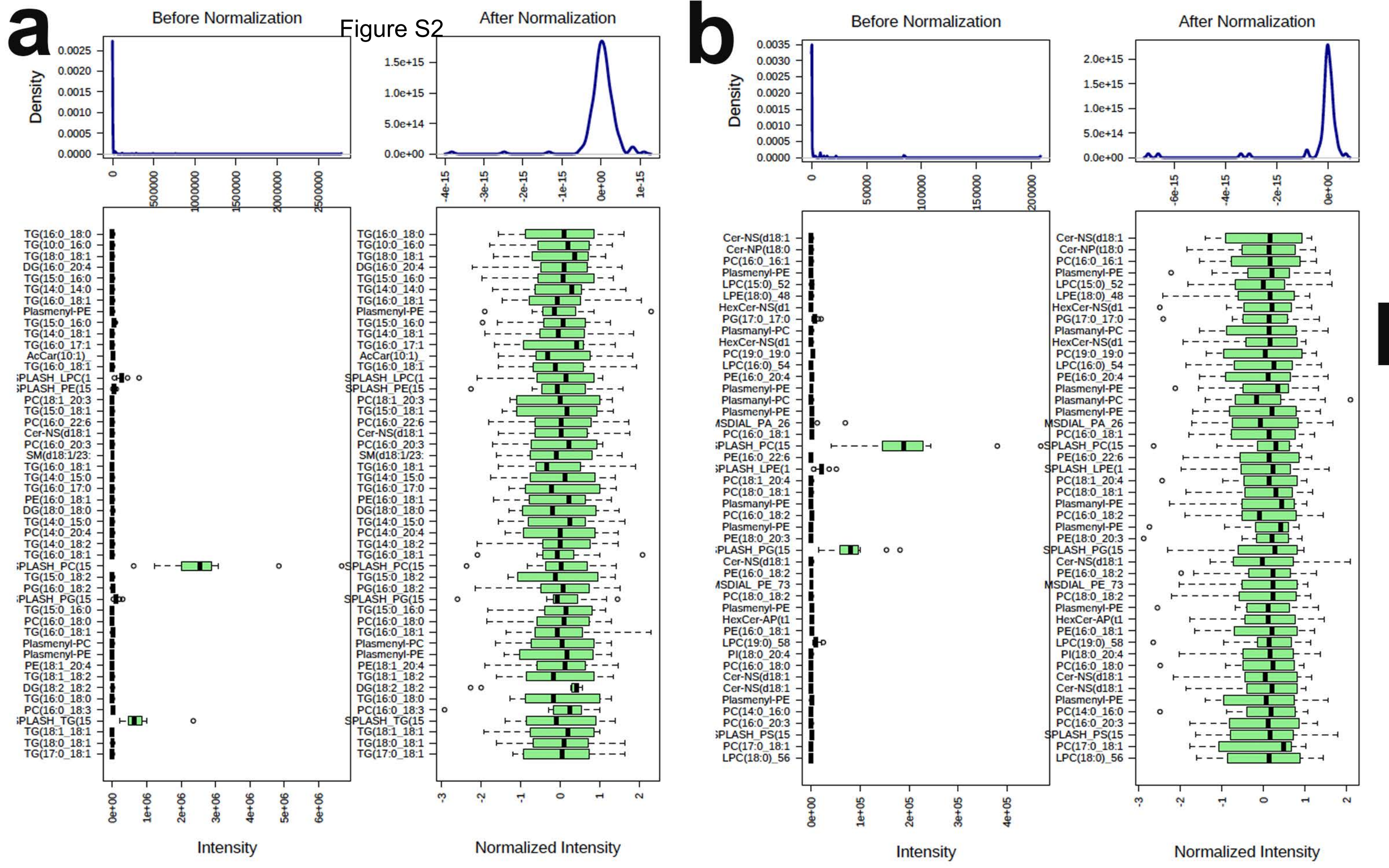
a
Before Normalization
After Normalization
Density
Intensity
Normalized Intensity
TG(16:0_18:0
TG(10:0_16:0
TG(18:0_18:1
DG(16:0_20:4
TG(15:0_16:0
TG(14:0_14:0
TG(16:0_18:1
Plasmenyl-PE
TG(15:0_16:0
TG(14:0_18:1
TG(16:0_17:1
AcCar(10:1)_
TG(16:0_18:1
SPLASH_LPC(1
SPLASH_PE(15
PC(18:1_20:3
TG(15:0_18:1
PC(16:0_22:6
Cer-NS(d18:1
PC(16:0_20:3
SM(d18:1/23:
TG(16:0_18:1
TG(14:0_15:0
TG(16:0_17:0
PE(16:0_18:1
DG(18:0_18:0
TG(14:0_15:0
PC(14:0_20:4
TG(14:0_18:2
TG(16:0_18:1
SPLASH_PC(15
TG(15:0_18:2
PG(16:0_18:2
SPLASH_PG(15
TG(15:0_16:0
PC(16:0_18:0
TG(16:0_18:1
Plasmenyl-PC
Plasmenyl-PE
PE(18:1_20:4
TG(18:1_18:2
DG(18:2_18:2
TG(16:0_18:0
PC(16:0_18:3
SPLASH_TG(15
TG(18:1_18:1
TG(18:0_18:1
TG(17:0_18:1
b
Before Normalization
After Normalization
Density
Intensity
Normalized Intensity
Cer-NS(d18:1
Cer-NP(t18:0
PC(16:0_16:1
Plasmenyl-PE
LPC(15:0)_52
LPE(18:0)_48
HexCer-NS(d1
PG(17:0_17:0
Plasmanyl-PC
HexCer-NS(d1
PC(19:0_19:0
LPC(16:0)_54
PE(16:0_20:4
Plasmenyl-PE
Plasmanyl-PC
Plasmenyl-PE
MSDIAL_PA_26
PC(16:0_18:1
SPLASH_PC(15
PE(16:0_22:6
SPLASH_LPE(1
PC(18:1_20:4
PC(18:0_18:1
Plasmanyl-PE
PC(16:0_18:2
Plasmenyl-PE
PE(18:0_20:3
SPLASH_PG(15
Cer-NS(d18:1
PE(16:0_18:2
MSDIAL_PE_73
PC(18:0_18:2
Plasmenyl-PE
HexCer-AP(t1
PE(16:0_18:1
LPC(19:0)_58
PI(18:0_20:4
PC(16:0_18:0
Cer-NS(d18:1
Cer-NS(d18:1
Plasmenyl-PE
PC(14:0_16:0
PC(16:0_20:3
SPLASH_PS(15
PC(17:0_18:1
LPC(18:0)_56

Figure S3

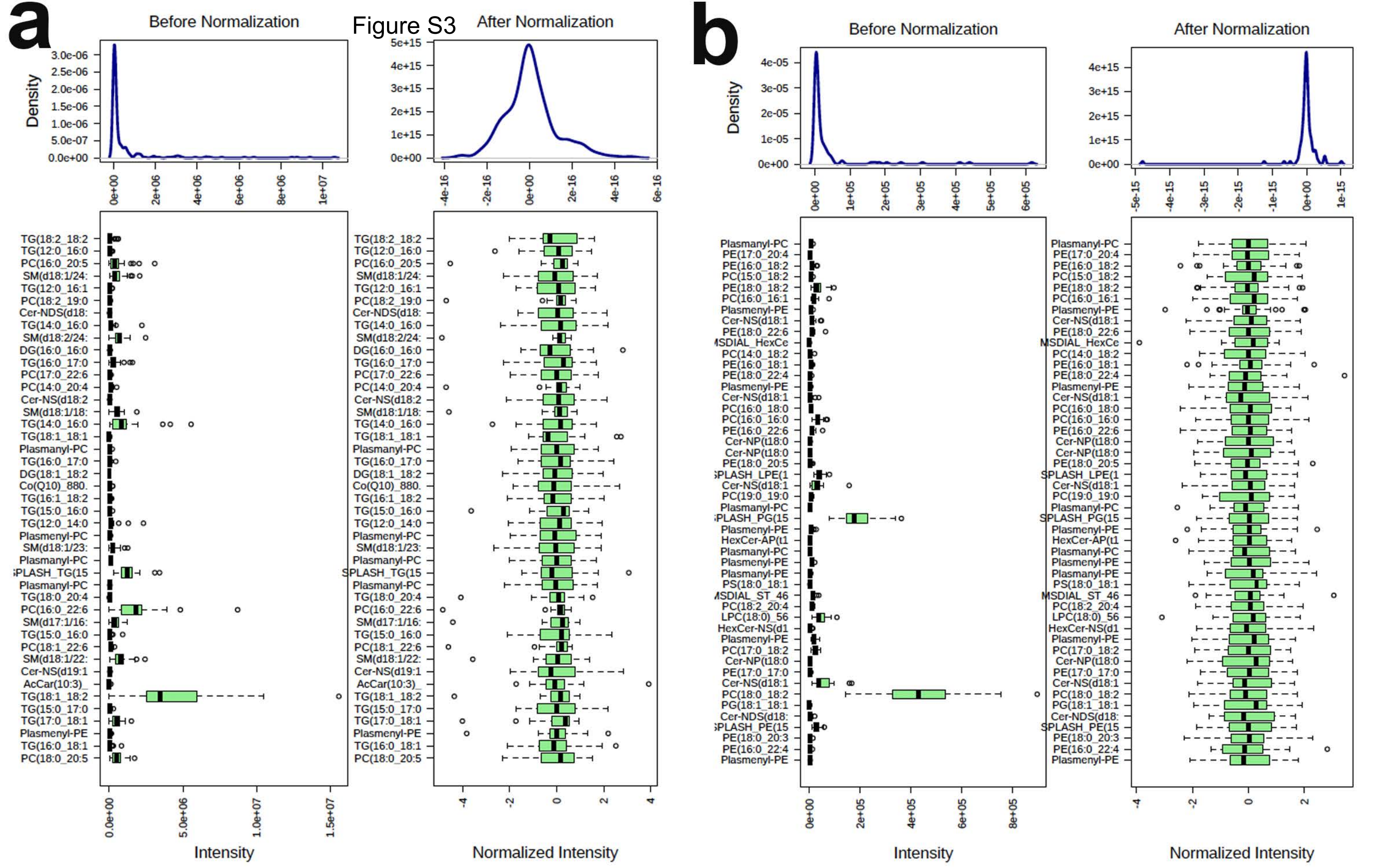

a
Before Normalization
After Normalization
Density
Intensity
Normalized Intensity
TG(18:2_18:2
TG(12:0_16:0
PC(16:0_20:5
SM(d18:1/24:
TG(12:0_16:1
PC(18:2_19:0
Cer-NDS(d18:
TG(14:0_16:0
SM(d18:2/24:
DG(16:0_16:0
TG(16:0_17:0
PC(17:0_22:6
PC(14:0_20:4
Cer-NS(d18:2
SM(d18:1/18:
TG(14:0_16:0
TG(18:1_18:1
Plasmanyl-PC
TG(16:0_17:0
DG(18:1_18:2
Co(Q10)_880.
TG(16:1_18:2
TG(15:0_16:0
TG(12:0_14:0
Plasmenyl-PC
SM(d18:1/23:
Plasmanyl-PC
SPLASH_TG(15
Plasmanyl-PC
TG(18:0_20:4
PC(16:0_22:6
SM(d17:1/16:
TG(15:0_16:0
PC(18:1_22:6
SM(d18:1/22:
Cer-NS(d19:1
AcCar(10:3)_
TG(18:1_18:2
TG(15:0_17:0
TG(17:0_18:1
Plasmenyl-PE
TG(16:0_18:1
PC(18:0_20:5
b
Before Normalization
After Normalization
Density
Intensity
Normalized Intensity
Plasmanyl-PC
PE(17:0_20:4
PE(16:0_18:2
PC(15:0_18:2
PE(18:0_18:2
PC(16:0_16:1
Plasmenyl-PE
Cer-NS(d18:1
PE(18:0_22:6
MSDIAL_HexCe
PC(14:0_18:2
PE(16:0_18:1
PE(18:0_22:4
Plasmenyl-PE
Cer-NS(d18:1
PC(16:0_18:0
PC(16:0_16:0
PE(16:0_22:6
Cer-NP(t18:0
Cer-NP(t18:0
PE(18:0_20:5
SPLASH_LPE(1
Cer-NS(d18:1
PC(19:0_19:0
Plasmanyl-PC
SPLASH_PG(15
Plasmenyl-PE
HexCer-AP(t1
Plasmanyl-PC
Plasmenyl-PE
Plasmanyl-PE
PS(18:0_18:1
MSDIAL_ST_46
PC(18:2_20:4
LPC(18:0)_56
HexCer-NS(d1
Plasmenyl-PE
PC(17:0_18:2
Cer-NP(t18:0
PE(17:0_17:0
Cer-NS(d18:1
PC(18:0_18:2
PG(18:1_18:1
Cer-NDS(d18:
SPLASH_PE(15
PE(18:0_20:3
PE(16:0_22:4
Plasmenyl-PE

Figure S4

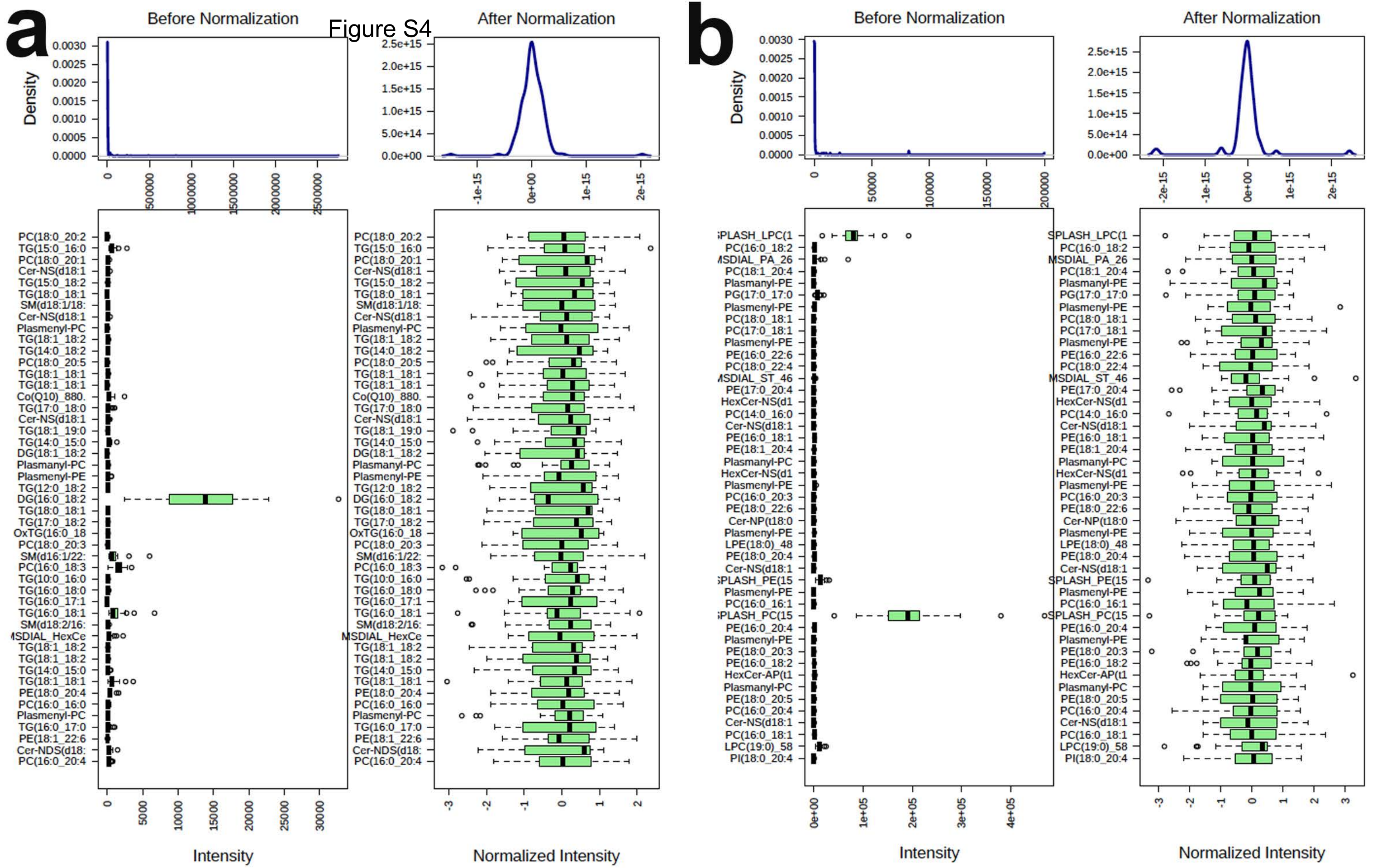

a
Before Normalization
After Normalization
Density
Intensity
Normalized Intensity
b
Before Normalization
After Normalization
Density
Intensity
Normalized Intensity

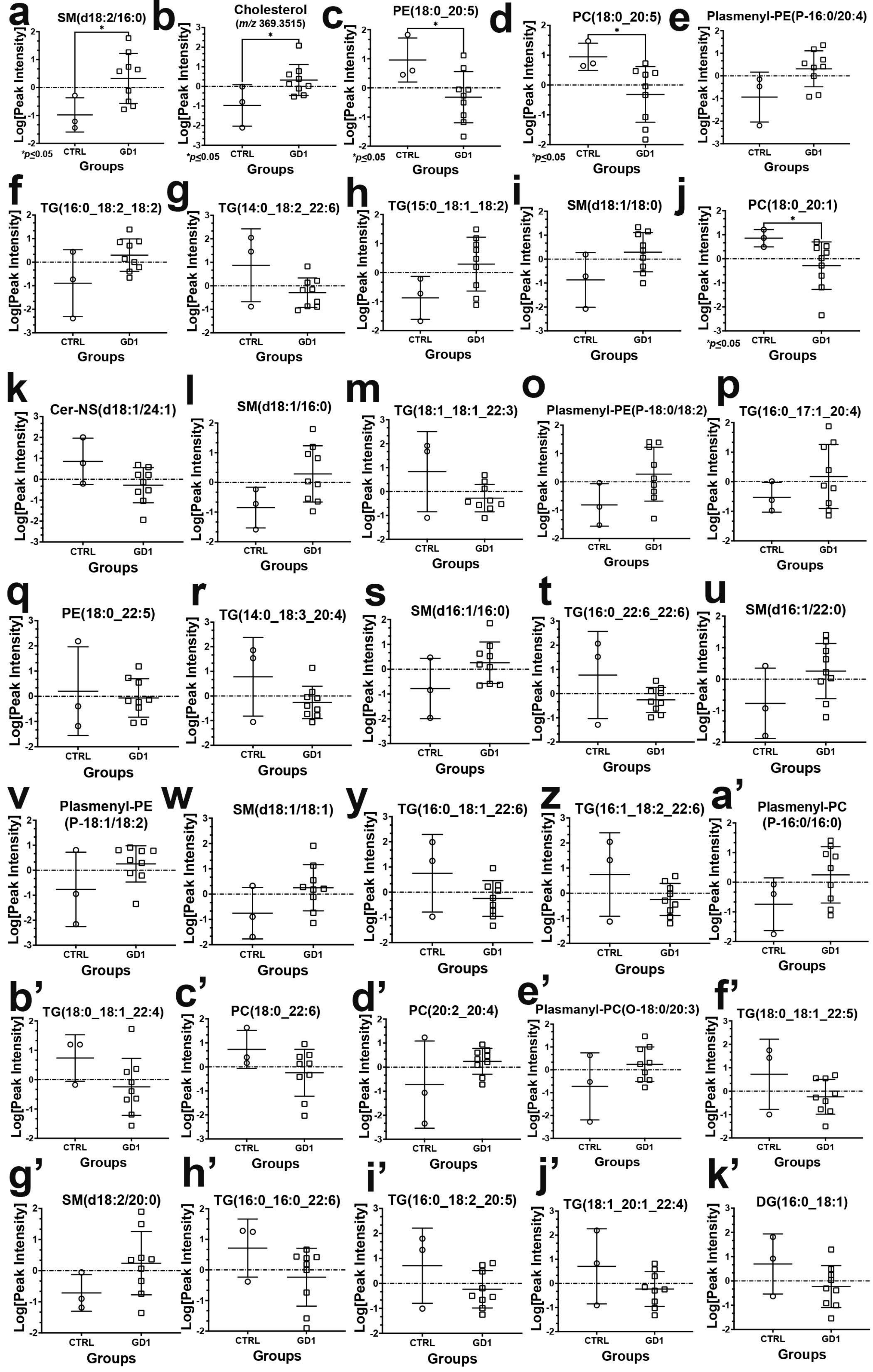

a SM(d18:2/16:0)
b Cholesterol (m/z 369.3515)
c PE(18:0_20:5)
d PC(18:0_20:5)
e Plasmenyl-PE(P-16:0/20:4)
f TG(16:0_18:2_18:2)
g TG(14:0_18:2_22:6)
h TG(15:0_18:1_18:2)
i SM(d18:1/18:0)
j PC(18:0_20:1)
k Cer-NS(d18:1/24:1)
l SM(d18:1/16:0)
m TG(18:1_18:1_22:3)
o Plasmenyl-PE(P-18:0/18:2)
p TG(16:0_17:1_20:4)
q PE(18:0_22:5)
r TG(14:0_18:3_20:4)
s SM(d16:1/16:0)
t TG(16:0_22:6_22:6)
u SM(d16:1/22:0)
v Plasmenyl-PE (P-18:1/18:2)
w SM(d18:1/18:1)
y TG(16:0_18:1_22:6)
z TG(16:1_18:2_22:6)
a' Plasmenyl-PC (P-16:0/16:0)
b' TG(18:0_18:1_22:4)
c' PC(18:0_22:6)
d' PC(20:2_20:4)
e' Plasmanyl-PC(O-18:0/20:3)
f' TG(18:0_18:1_22:5)
g' SM(d18:2/20:0)
h' TG(16:0_16:0_22:6)
i' TG(16:0_18:2_20:5)
j' TG(18:1_20:1_22:4)
k' DG(16:0_18:1)
Log[Peak Intensity]
Groups
CTRL
GD1
*p≤0.05

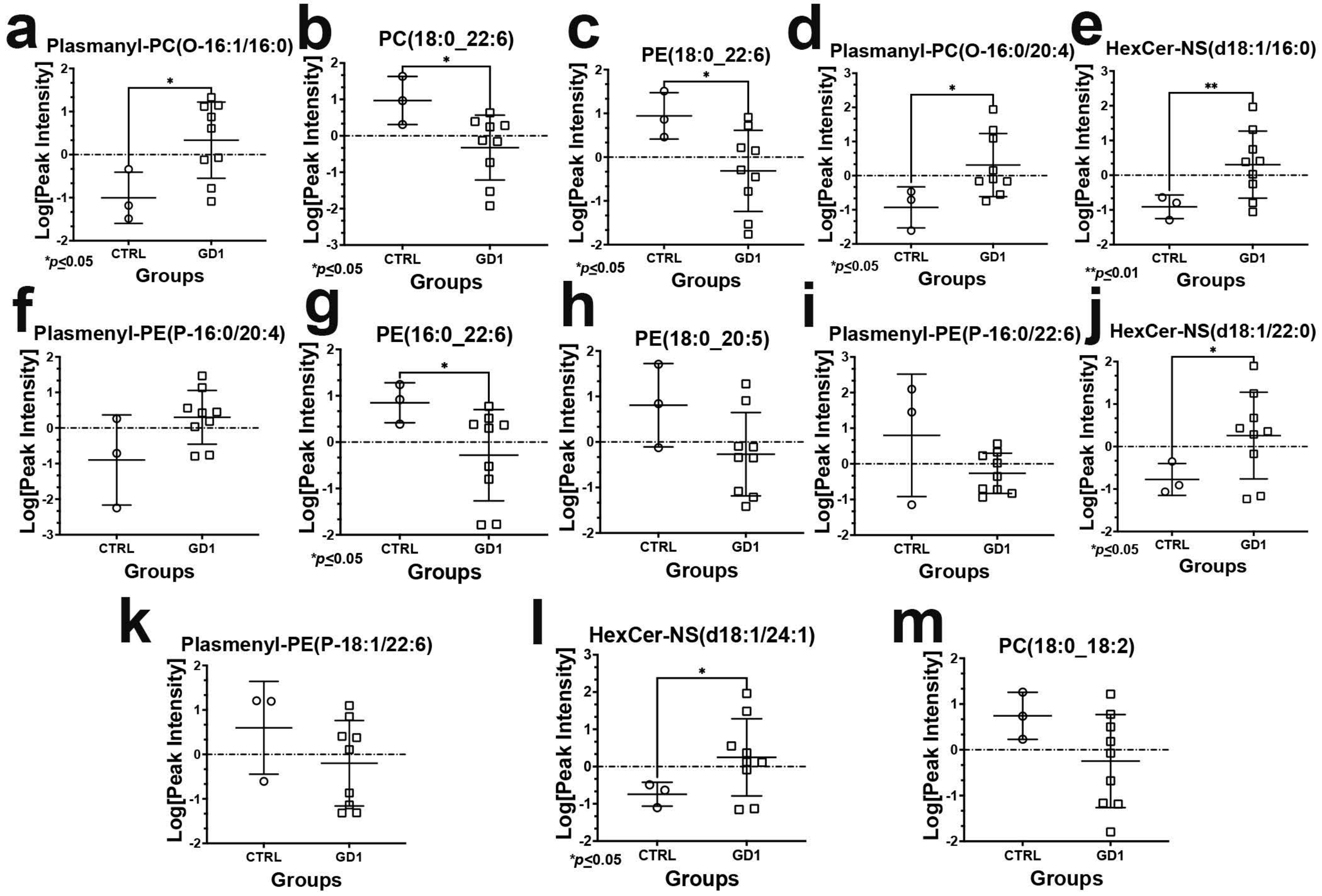

a
Plasmanyl-PC(O-16:1/16:0)
b
PC(18:0_22:6)
c
PE(18:0_22:6)
d
Plasmanyl-PC(O-16:0/20:4)
e
HexCer-NS(d18:1/16:0)
f
Plasmenyl-PE(P-16:0/20:4)
g
PE(16:0_22:6)
h
PE(18:0_20:5)
i
Plasmenyl-PE(P-16:0/22:6)
j
HexCer-NS(d18:1/22:0)
k
Plasmenyl-PE(P-18:1/22:6)
l
HexCer-NS(d18:1/24:1)
m
PC(18:0_18:2)
Log[Peak Intensity]
CTRL
GD1
Groups
*p≤0.05
**p≤0.01

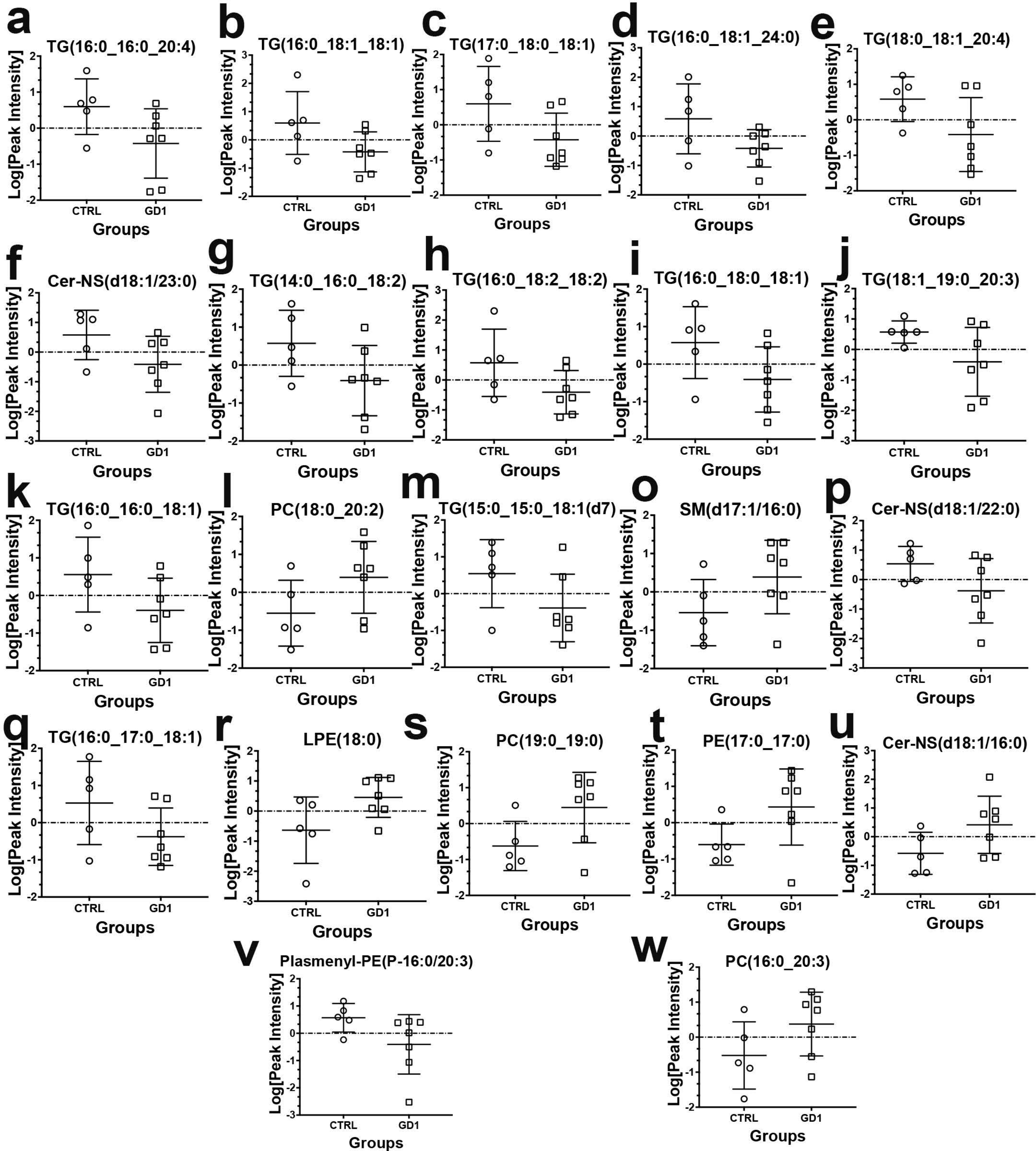

a
TG(16:0_16:0_20:4)
b
TG(16:0_18:1_18:1)
c
TG(17:0_18:0_18:1)
d
TG(16:0_18:1_24:0)
e
TG(18:0_18:1_20:4)
f
Cer-NS(d18:1/23:0)
g
TG(14:0_16:0_18:2)
h
TG(16:0_18:2_18:2)
i
TG(16:0_18:0_18:1)
j
TG(18:1_19:0_20:3)
k
TG(16:0_16:0_18:1)
l
PC(18:0_20:2)
m
TG(15:0_15:0_18:1(d7)
o
SM(d17:1/16:0)
p
Cer-NS(d18:1/22:0)
q
TG(16:0_17:0_18:1)
r
LPE(18:0)
s
PC(19:0_19:0)
t
PE(17:0_17:0)
u
Cer-NS(d18:1/16:0)
v
Plasmenyl-PE(P-16:0/20:3)
w
PC(16:0_20:3)
Log[Peak Intensity]
CTRL
GD1
Groups

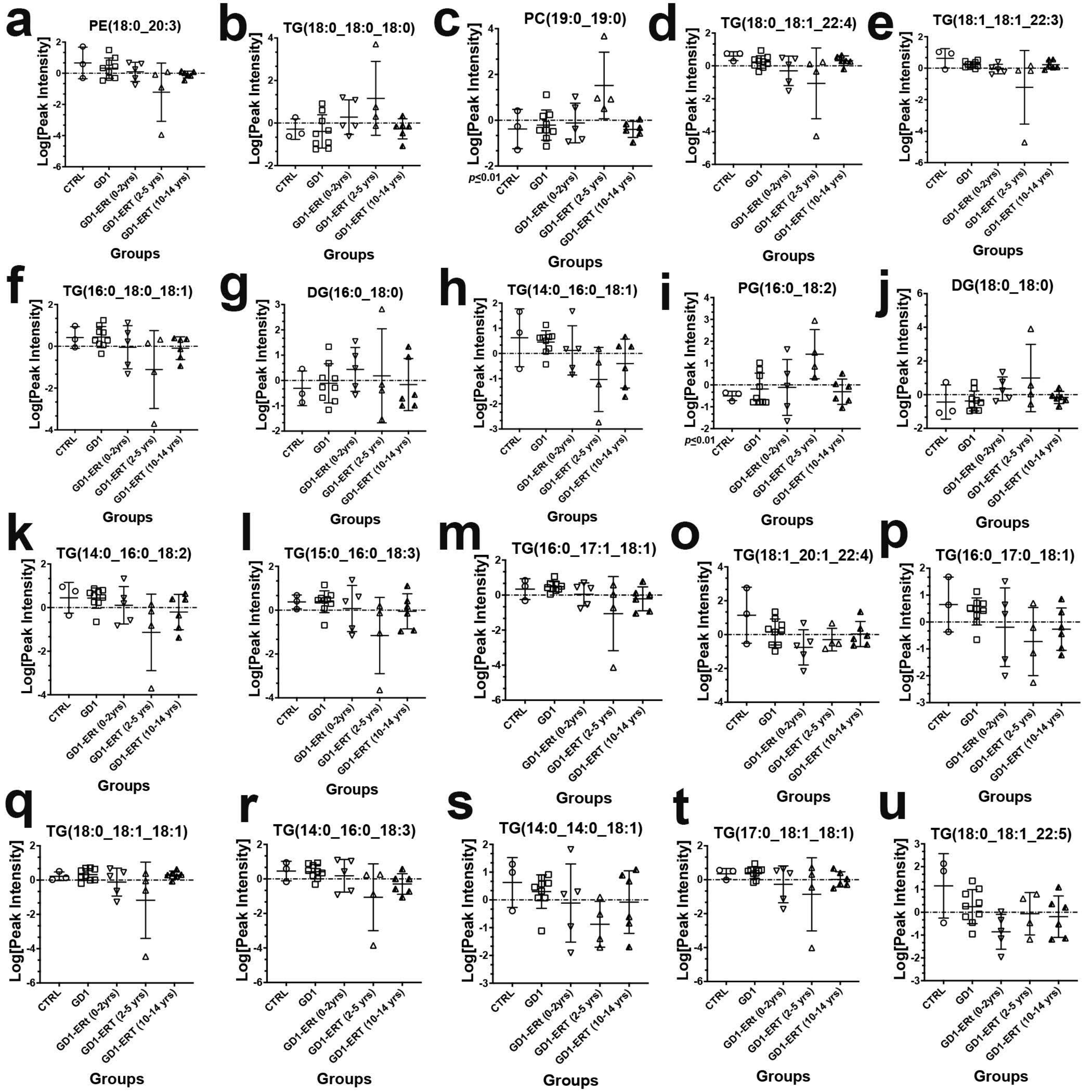

a
PE(18:0_20:3)
b
TG(18:0_18:0_18:0)
c
PC(19:0_19:0)
p≤0.01
d
TG(18:0_18:1_22:4)
e
TG(18:1_18:1_22:3)
f
TG(16:0_18:0_18:1)
g
DG(16:0_18:0)
h
TG(14:0_16:0_18:1)
i
PG(16:0_18:2)
p≤0.01
j
DG(18:0_18:0)
k
TG(14:0_16:0_18:2)
l
TG(15:0_16:0_18:3)
m
TG(16:0_17:1_18:1)
o
TG(18:1_20:1_22:4)
p
TG(16:0_17:0_18:1)
q
TG(18:0_18:1_18:1)
r
TG(14:0_16:0_18:3)
s
TG(14:0_14:0_18:1)
t
TG(17:0_18:1_18:1)
u
TG(18:0_18:1_22:5)
Log[Peak Intensity]
CTRL
GD1
GD1-ERt (0-2yrs)
GD1-ERT (2-5 yrs)
GD1-ERT (10-14 yrs)
Groups

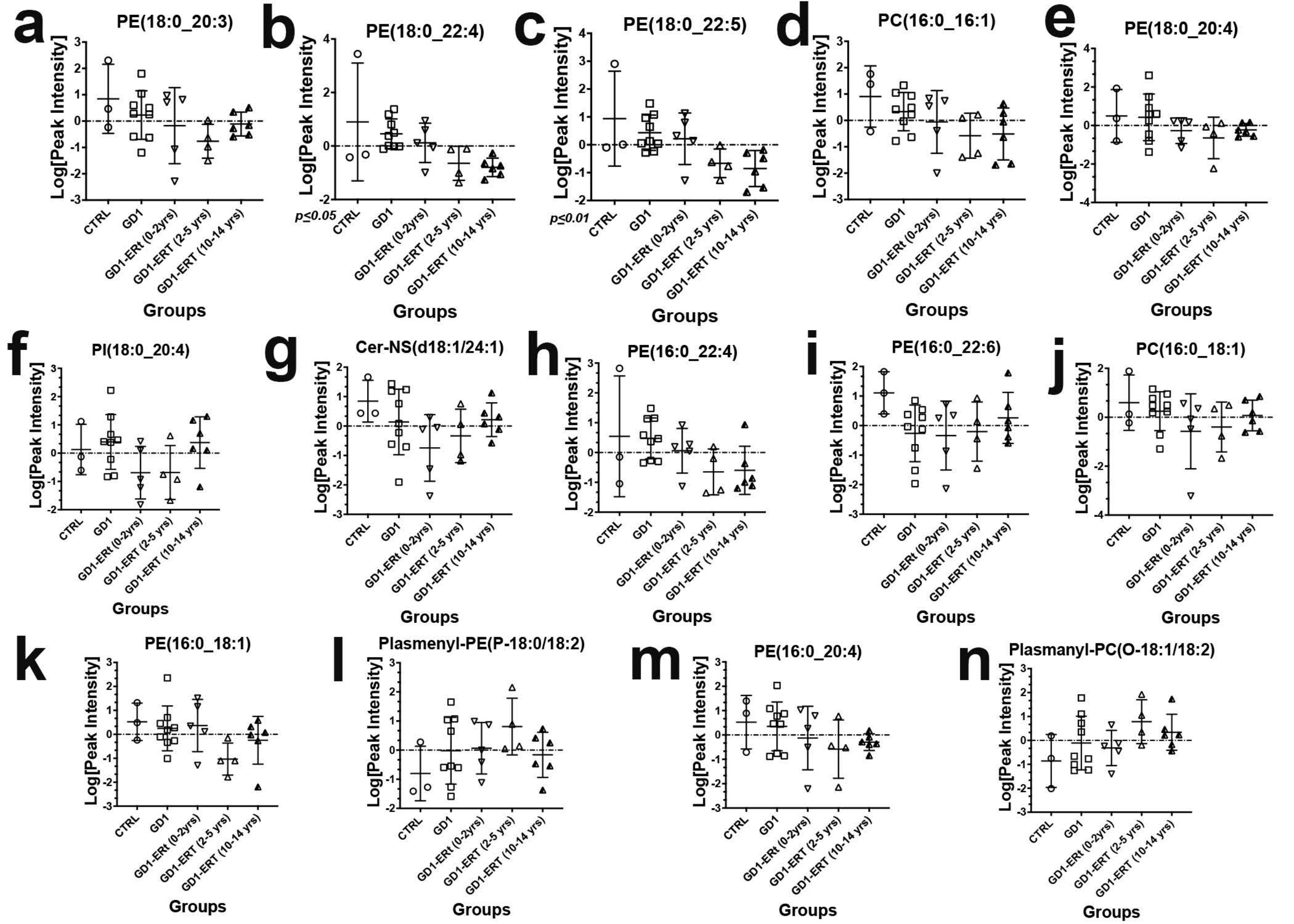
a
PE(18:0_20:3)
b
PE(18:0_22:4)
p≤0.05
c
PE(18:0_22:5)
p≤0.01
d
PC(16:0_16:1)
e
PE(18:0_20:4)
f
PI(18:0_20:4)
g
Cer-NS(d18:1/24:1)
h
PE(16:0_22:4)
i
PE(16:0_22:6)
j
PC(16:0_18:1)
k
PE(16:0_18:1)
l
Plasmenyl-PE(P-18:0/18:2)
m
PE(16:0_20:4)
n
Plasmanyl-PC(O-18:1/18:2)
Log[Peak Intensity]
CTRL
GD1
GD1-ERt (0-2yrs)
GD1-ERT (2-5 yrs)
GD1-ERT (10-14 yrs)
Groups

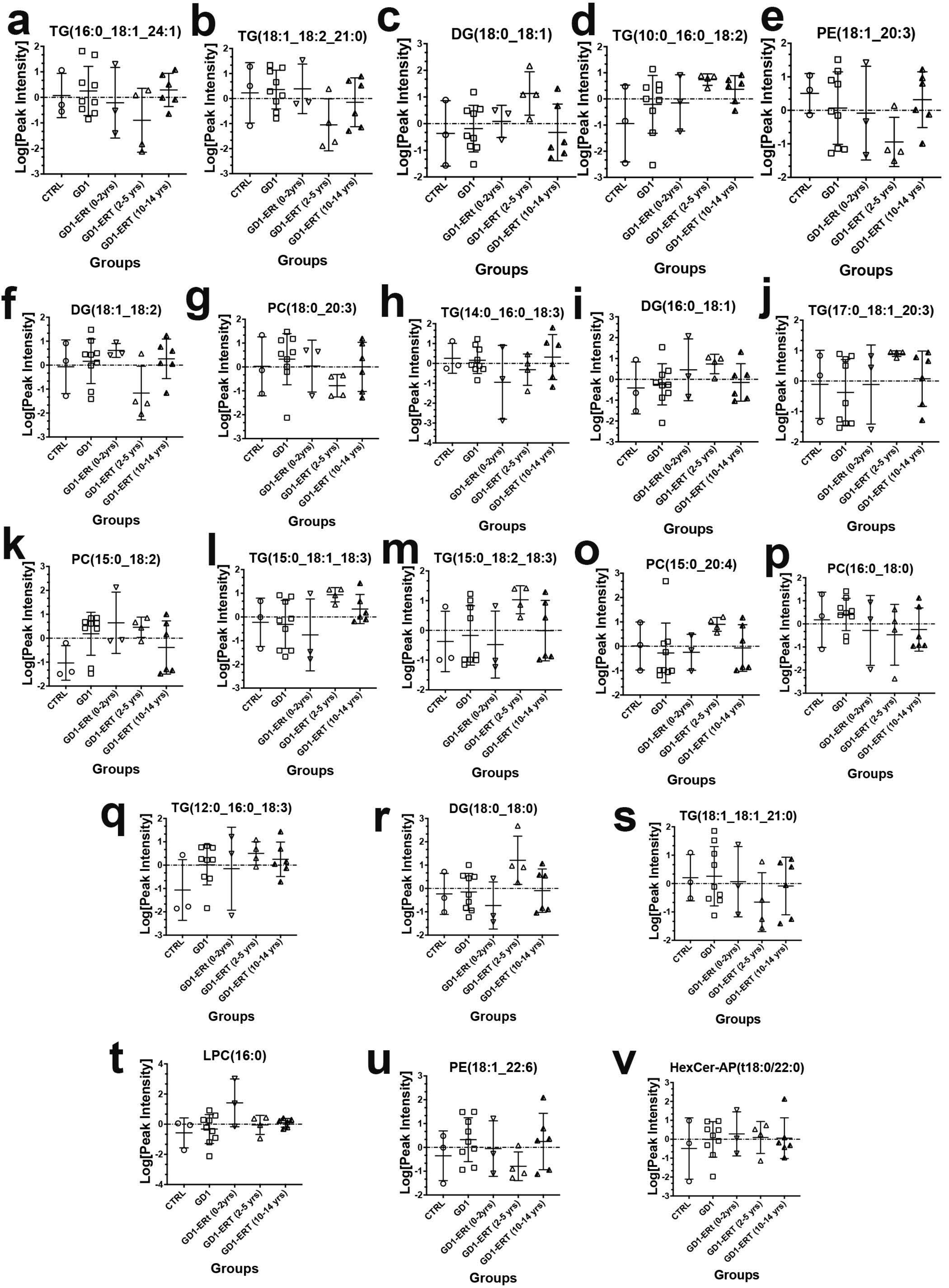
a
TG(16:0_18:1_24:1)
b
TG(18:1_18:2_21:0)
c
DG(18:0_18:1)
d
TG(10:0_16:0_18:2)
e
PE(18:1_20:3)
f
DG(18:1_18:2)
g
PC(18:0_20:3)
h
TG(14:0_16:0_18:3)
i
DG(16:0_18:1)
j
TG(17:0_18:1_20:3)
k
PC(15:0_18:2)
l
TG(15:0_18:1_18:3)
m
TG(15:0_18:2_18:3)
o
PC(15:0_20:4)
p
PC(16:0_18:0)
q
TG(12:0_16:0_18:3)
r
DG(18:0_18:0)
s
TG(18:1_18:1_21:0)
t
LPC(16:0)
u
PE(18:1_22:6)
v
HexCer-AP(t18:0/22:0)
Log[Peak Intensity]
CTRL
GD1
GD1-ERt (0-2yrs)
GD1-ERT (2-5 yrs)
GD1-ERT (10-14 yrs)
Groups

Table S1. Demographics of Gaucher disease patients.

Subjects#	*GBA1* genotype	Age at Dx	Gender	Follow-up/Treatment	Symptoms
**330/1**	c.1226A>G(N409S; *aka* N370S)/ c.1226A>G(N409S; *aka* N370S)	55.9 yrs	F	No GD-I specific treatment	Hepatosplenomegaly, hematological alterations, skeletal manifestations
**166/2**	c.1226A>G(N409S; *aka* N370S)/ c.1226A>G(N409S; *aka* N370S)	47.3 yrs	F	ERT (velaglucerase alfa)	Hepatosplenomegaly, hematological alterations, skeletal manifestations
**744/3**	c.1226A>G(N409S; *aka* N370S)/ c.1226A>G(N409S; *aka* N370S)	39.45 yrs	M	No GD-I specific treatment	None
**463/4**	c.1448T>C(L483P; *aka* L444P)/?	16.5 yrs	M	No GD-I specific treatment	None
**318/5**	c.1226A>G(N409S; *aka* N370S)/ c.1226A>G(N409S; *aka* N370S)	42.86 yrs	F	SRT (eliglustat)	Visceromegaly and Hematological
**430/6**	c.680A>G (p.N227S; *aka* N188S)/ c.1448T>C(L483P; *aka* L444P)	49.82 yrs	F	ERT (tagliglucerase alfa)	Skeletal, Visceromegaly and Hematological
**600/7**	c.1342G>C (p.D448H; aka D409H)/ c.1448T>C(L483P; *aka* L444P)	73.45 yrs	F	No GD-I specific treatment	Parkinsonism
**464/8**	c.1226A>G(N409S; *aka* N370S)/ c.1085C>T(p.T362I; *aka* T323I)	5.81 yrs	F	ERT (imiglucerase)	Hepatosplenomegaly, hematological alterations and slow growth
**222/9**	c.1226A>G(N409S; *aka* N370S)/?	74.1 yrs	F	ERT (velaglucerase alfa)	Hepatosplenomegaly, hematological alterations, skeletal manifestations
**159/10**	c.1226A>G(N409S; *aka* N370S)/ c.1226A>G(N409S; *aka* N370S)	54.89 yrs	M	ERT (velaglucerase alfa)	Hepatosplenomegaly, hematological alterations, skeletal manifestations
**332/11**	c.1226A>G(N409S; *aka* N370S)/ c.1035insG	45.6 yrs	F	ERT (velaglucerase alfa)	Hepatosplenomegaly, hematological alterations, parkinsonism
**601/12**	c.1226A>G(N409S; *aka* N370S)/?	65.3 yrs	M	ERT (velaglucerase alfa)	Hepatosplenomegaly, hematological alterations.
**919/13**	c.1226A>G(N409S; *aka* N370S)/ c.1226A>G(N409S; *aka* N370S)	55.94 yrs	F	ERT (imiglucerase)	Hepatosplenomegaly, hematological alterations.
**918/14**	c.1226A>G(N409S; *aka* N370S)/ c.1226A>G(N409S; *aka* N370S)	60.33 yrs	M	ERT (imiglucerase)	Hepatosplenomegaly, hematological alterations.
**176/15**	c.1226A>G(N409S; *aka* N370S)/ c.1226A>G(N409S; *aka* N370S)	32.71 yrs	M	ERT (imiglucerase)	Skeletal disease, malaise, hematological alterations.

Dx, diagnosis; ERT, enzyme replacement therapy (ERT); GD-I, Gaucher disease type I; Yrs, years.

Table S2. Analysis and identification of lipids from serum-derived EVs of GD1 subjects.

Lipid Species	*m/z*-r.t.	*t* (stat)	*p* (value)	-log10(p)
**Positive-ion mode**				
PE(18:0_22:6)	792.5537-7.97	2.719	0.021599*	1.6656
SM(d18:1/17:0)	717.5915-7.62	-2.6758	0.023259*	1.6334
SM(d18:2/16:0)	701.5595-6.81	-2.3204	0.042745*	1.3691
Cholesterol	369.3515-14.51	-2.2829	0.045557*	1.3414
PE(18:0_20:5)	766.5379-7.75	2.2389	0.04912*	1.3089
PC(18:0_20:5)	808.5842-7.64	2.1948	0.052904	1.2765
Plasmenyl-PE(P-16:0/20:4)	724.5271-7.81	-2.1656	0.055586	1.255
TG(16:0_18:2_18:2)	872.7705-13.90	-2.0237	0.070537	1.1516
TG(14:0_18:2_22:6)	892.7392-12.71	1.9618	0.078203	1.1068
TG(15:0_18:1_18:2)	860.7702-14.14	-1.9523	0.079448	1.0999
SM(d18:1/18:0)	731.6064-7.95	-1.9456	0.080332	1.0951
PC(18:0_20:1)	816.6457-9.31	1.9079	0.085499	1.068
Cer-NS(d18:1/24:1)	648.6291-10.14	1.9004	0.086559	1.0627
SM(d18:1/16:0)	703.5749-7.32	-1.8891	0.088193	1.0546
TG(18:1_18:1_22:3)	954.8469-14.94	1.828	0.097492	1.011
**Negative-ion Mode**				
PC(16:0_20:4)	826.5612-7.44	-3.1337	0.010624*	1.9737
PC(16:0_22:5)	852.5763-7.63	2.7096	0.021949*	1.6586
Plasmanyl-PC(O-16:1/16:0)	762.5676-8.27	-2.404	0.037061*	1.4311
PC(18:0_22:6)	878.5925-7.86	2.2806	0.045736*	1.3397
PE(18:0_22:6)	790.5401-8.00	2.1853	0.053762	1.2695
Plasmanyl-PC(O-16:0/20:4)	812.5818-7.78	-2.1399	0.058048	1.2362
HexCer-NS(d18:1/16:0)	744.5632-7.52	-2.0761	0.064623	1.1896
Plasmenyl-PE(P-16:0/20:4)	722.5140-7.84	-2.0408	0.068557	1.1639
PE(16:0_22:6)	762.5085-7.41	1.8823	0.089189	1.0497

Cer-NS, ceramide non-hydroxy-fatty acid sphingosine; GD1, Gaucher disease type 1; HexCer-NS, hexosylceramide non-hydroxy fatty acid-sphingosine; PC, phosphatidylcholine; PE, phosphoethanolamine; r.t., retention time; SM, sphingomyelin; TG, triglyceride. *$p<0.05$

Table S3. Analysis and identification of lipids from urine-derived EVs of GD1 subjects.

**Lipid Species**	***m/z*-r.t.**	***t* (stat)**	***p* (value)**	**-log10(p)**
**Positive-ion mode**				
TG(16:0_18:1_22:0)	934.8796-15.82	2.4433	0.03465*	1.4603
TG(12:0_14:0_16:0)	740.6760-13.04	2.2816	0.045659*	1.3405
TG(16:0_16:0_20:4)	872.7707-14.20	1.9583	0.078656	1.1043
TG(16:0_18:1_18:1)	876.8018-14.75	1.9569	0.078845	1.1032
TG(17:0_18:0_18:1)	892.8326-15.31	1.9531	0.079334	1.1005
TG(16:0_18:1_24:0)	962.9088-16.09	1.9037	0.086095	1.065
TG(18:0_18:1_20:4)	926.8162-14.70	1.8912	0.087894	1.056
Cer-NS(d18:1/23:0)	636.6295-10.72	1.8744	0.090348	1.0441
TG(14:0_16:0_18:2)	820.7386-13.84	1.854	0.093426	1.0295
TG(16:0_18:2_18:2)	872.7705-13.90	1.8538	0.093466	1.0293
TG(16:0_18:0_18:1)	878.8159-15.16	1.8519	0.093753	1.028
TG(18:1_19:0_20:3)	942.8486-15.08	1.8465	0.094588	1.0242
**Negative-ion Mode**				
LPC(15:0)	526.3153-0.91	3.0483	0.012288*	1.9105
HexCer-NS(d18:1/16:0)	744.5632-7.52	-2.424	0.035813*	1.446
LPE(18:0)	480.3100-2.26	-2.1475	0.05731	1.2418
PC(19:0_19:0)	862.6564-10.15	-2.0902	0.063109	1.1999
PE(17:0_17:0)	718.5406-8.83	-1.9916	0.074424	1.1283
Cer-NS(d18:1/16:0)	582.5107-7.99	-1.8808	0.089402	1.0487
Plasmenyl-PE(P-16:0/20:3)	724.5293-8.11	1.839	0.095749	1.0189

Cer-NS, ceramide non-hydroxy-fatty acid sphingosine; GD1, Gaucher disease type 1; HexCer-NS, hexosylceramide non-hydroxy fatty acid sphingosine; LPC, lysophosphatidylcholine; LPE, lysophosphatidylethanolamine; PC, phosphatidylcholine; PE, phosphoethanolamine; r.t., retention time; SM, sphingomyelin; TG, triglyceride. *$p<0.05$

Table S4. Fold-change analysis of serum EV-isolated lipids from GD1 patients in positive-ion and negative-ion modes

Lipid Species	*m/z*-r.t.	Fold Change	log2(FC)
**Positive-ion mode**			
TG(16:0_22:6_22:6)	968.7695-12.97	26.038	4.7026
TG(14:0_18:2_22:6)	892.7392-12.71	7.4507	2.8974
DG(18:0_18:0)	642.6035-10.85	6.9102	2.7887
TG(18:2_18:3_18:3)	892.7389-12.23	6.6478	2.7329
TG(18:2_18:2_18:3)	894.7540-12.88	6.0647	2.6004
TG(16:1_18:2_22:6)	918.7543-12.94	5.3847	2.4289
TG(14:0_18:3_20:4)	866.7237-12.58	5.0654	2.3407
TG(16:1_18:2_18:4)	866.7233-12.11	4.7132	2.2367
TG(18:2_18:2_20:5)	918.7541-12.68	2.8781	1.5251
PE(40:4)	796.5841-8.62	2.87	1.5211
TG(16:0_18:2_22:6)	920.7694-13.47	2.7878	1.4791
TG(18:1_18:2_22:0)	958.8796-15.55	2.6432	1.4023
PE(16:0_22:4)	768.5527-7.97	2.573	1.3635
TG(14:0_18:2_18:4)	840.7076-12.11	2.5455	1.348
TG(12:0_14:0_16:0)	740.6760-13.04	2.5412	1.3455
TG(12:0_18:2_18:2)	816.7073-12.52	2.5205	1.3337
TG(14:0_18:2_20:5)	866.7233-12.33	2.4464	1.2907
OxTG(16:0_16:0_18:2)	862.7473-13.55	2.3313	1.2211
PE(18:0_20:5)	766.5379-7.75	2.1873	1.1291
TG(12:0_14:0_18:1)	766.6923-13.06	2.1872	1.1291
PC(16:0_20:5)	780.5543-7.01	2.1254	1.0877
PE(18:0_22:5)	794.5698-8.18	2.0599	1.0426
TG(16:0_18:2_20:5)	894.7548-13.30	2.0557	1.0396
TG(16:0_18:1_22:6)	922.7848-13.97	2.0496	1.0353
DG(16:0_18:1)	612.5564-9.13	2.0162	1.0116
DG(16:0_18:2)	610.5406-8.74	0.49666	-1.0097
AcCar(10:3)	310.1990-0.32	0.42454	-1.236
PG(16:0_18:2)	764.5447-7.33	0.25428	-1.9755
**Negative-ion Mode**			
PE(18:0_22:4)	794.5716-8.64	3.0845	1.625
PE(18:0_22:5)	792.5557-8.42	2.9471	1.5593
PE(16:0_22:4)	766.5403-8.00	2.7499	1.4594
Cer-NDS(d18:0/24:0)	696.6527-11.80	2.7371	1.4527
PE(18:0_20:5)	764.5245-7.78	2.1893	1.1305
PE(18:0_22:6)	790.5401-8.00	2.1642	1.1138
PC(18:0_22:4)	882.6243-8.47	2.1555	1.108
HexCer-NS(d18:1/16:0)	744.5632-7.52	0.46941	-1.0911
HexCer-NS(d18:1/22:0)	828.6587-9.63	0.41333	-1.2746
HexCer-NS(d18:1/24:1)	854.6746-9.55	0.35045	-1.5127
PG(18:1_18:1)	773.5358-7.46	0.13575	-2.881

AcCar, acylcarnitine; DG, diacylglycerol; Cer-NDS, ceramide non-dihydroxy-fatty acid sphingosine; GD1, Gaucher disease type 1; HexCer-NS, hexosylceramide non-hydroxy fatty acid sphingosine; OxTG, oxidized triglyceride; PC, phosphatidylcholine; PE, phosphoethanolamine; PG, phosphatidylglycerol; r.t., retention time; SM, sphingomyelin; TG, triglyceride.

Table S5. Fold-change analysis of urine EV-isolated lipids in positive-ion and negative-ion modes.

Lipid Species	*m/z*-r.t.	Fold Change	log2(FC)
**Positive-ion mode**			
TG(18:1_22:0_22:0)	1,018.9726-16.57	17.385	4.1198
TG(18:0_18:1_24:0)	990.9408-16.35	6.0466	2.5961
Cer-NS(d18:1/23:0)	636.6295-10.72	5.1721	2.3708
TG(16:0_18:1_24:0)	962.9088-16.09	4.1454	2.0515
TG(17:0_18:0_18:1)	892.8326-15.31	3.9753	1.991
TG(16:0_18:1_23:0)	948.8930-15.93	3.6344	1.8617
PC(14:0_20:4)	754.5380-6.82	3.4285	1.7776
TG(14:0_18:2_18:2)	844.7391-13.30	3.4054	1.7678
TG(17:0_18:2_18:2)	886.7844-14.17	3.4047	1.7675
TG(16:0_18:1_21:0)	920.8640-15.64	3.2791	1.7133
TG(16:1_18:2_18:2)	870.7549-13.43	3.1948	1.6757
Cer-NS(d19:1/24:0)	664.6608-11.69	3.0262	1.5975
TG(14:0_14:1_16:1)	764.6744-12.52	2.928	1.5499
TG(16:0_18:1_22:0)	934.8796-15.82	2.8425	1.5072
TG(16:0_16:0_18:4)	844.7393-13.58	2.8013	1.4861
DG(18:0_18:0)	642.6035-10.85	2.7996	1.4852
DG(18:0_18:1)	640.5877-9.92	2.7943	1.4825
PC(15:0_22:6)	792.5518-6.95	2.7508	1.4599
PC(18:0_20:1)	816.6457-9.31	2.7365	1.4523
DG(18:1_18:2)	636.5568-8.82	2.7286	1.4482
TG(18:1_18:2_19:0)	916.8317-14.96	2.6753	1.4197
TG(16:0_17:0_18:1)	864.8016-14.95	2.6731	1.4185
Cer-NDS(d18:0/24:0)	652.6602-11.78	2.6318	1.3961
TG(15:0_17:0_17:0)	838.7846-13.74	2.621	1.3901
TG(16:0_18:1_20:2)	902.8175-16.15	2.5635	1.3581
TG(15:0_18:2_18:2)	858.7541-13.65	2.5586	1.3554
TG(16:0_17:1_20:4)	884.7708-13.94	2.5447	1.3475
PE(16:0_22:4)	768.5527-7.97	2.5421	1.346
PC(18:2_19:0)	800.6155-8.42	2.534	1.3414
TG(10:0_16:0_18:2)	764.6746-12.31	2.5022	1.3232
TG(18:0_18:1_20:4)	926.8162-14.70	2.4669	1.3027
PC(18:2_20:4)	806.5685-6.99	2.4172	1.2733
Plasmanyl-PC(O-20:0/20:4)	824.6536-9.11	2.4164	1.2728
TG(16:0_17:0_18:0)	866.8174-15.33	2.3994	1.2627
TG(12:0_18:2_18:2)	816.7073-12.52	2.3826	1.2525
TG(16:0_18:0_24:0)	964.9262-16.35	2.3818	1.2521
TG(16:0_18:2_18:2)	872.7705-13.90	2.359	1.2382
DG(16:0_18:0)	614.5724-9.88	2.3447	1.2294
TG(18:0_18:0_18:0)	908.8641-15.97	2.3304	1.2206
Cer-NS(d18:1/22:0)	622.6132-10.22	2.3253	1.2174

PC(14:0_16:1)	704.5217-6.82	2.3058	1.2053
TG(16:0_18:0_18:1)	878.8159-15.16	2.292	1.1966
TG(16:0_18:0_18:0)	880.8328-15.52	2.2712	1.1835
Cer-NS(d18:1/24:0)	650.6448-11.30	2.2322	1.1585
TG(14:0_16:0_18:4)	816.7073-12.90	2.19	1.1309
TG(12:0_14:0_18:1)	766.6923-13.06	2.1669	1.1157
TG(16:0_16:0_18:0)	852.8007-15.18	2.1628	1.1129
DG(16:0_18:1)	612.5564-9.13	2.129	1.0902
TG(16:0_18:0_20:0)	908.8637-15.83	2.1101	1.0773
TG(17:0_18:1_20:3)	914.8162-14.62	2.0918	1.0648
TG(18:1_18:1_23:0)	974.9101-15.96	2.0845	1.0597
TG(16:0_18:1_18:1)	876.8018-14.75	2.0201	1.0144
PC(17:0_22:6)	820.5828-7.52	2.0195	1.014
Cer-NS(d16:1/22:0)	594.5822-9.39	2.0127	1.0091
TG(18:0_18:1_20:3)	928.8319-14.84	2.0088	1.0063
TG(14:0_18:1_18:2)	846.7545-13.84	2.0027	1.0019
TG(18:1_18:1_24:1)	986.9111-15.84	0.47502	-1.0739
PC(18:0_20:5)	808.5842-7.64	0.35297	-1.5024
AcCar(10:1)	314.2322-0.35	0.33716	-1.5685
PC(18:0_20:2)	814.6318-8.69	0.18898	-2.4037
Plasmanyl-PC(O-16:0/16:0)	720.5908-8.34	0.111	-3.1714
**Negative-ion Mode**			
PE(16:0_22:4)	766.5403-8.00	2.4843	1.3128
PC(18:2_18:2)	826.5609-7.15	0.48158	-1.0542
Cer-NS(d18:1/20:0)	638.5732-9.42	0.472	-1.0831
Cer-NS(d18:1/16:0)	582.5107-7.99	0.39283	-1.348
Cer-NS(d18:1/24:1)	692.6207-10.19	0.35693	-1.4863
Plasmanyl-PC(O-16:1/18:1)	788.5824-8.30	0.34683	-1.5277
Cer-NS(d18:1/18:0)	610.5418-8.69	0.32179	-1.6358
LPE(18:0)	480.3100-2.26	0.25201	-1.9885
Plasmanyl-PC(O-16:0/16:0)	764.5831-8.37	0.063372	-3.98

AcCar, acylcarnitine; DG, diacylglycerol; Cer-NDS, ceramide non-dihydroxy fatty acid sphingosine; Cer-NS, ceramide non-hydroxy fatty acid sphingosine; GD1, Gaucher disease type 1; HexCer-NS, hexosylceramide non-hydroxy fatty acid sphingosine; LPE, lysophosphatidylethanolamine; OxTG, oxidized triglyceride; PC, phosphatidylcholine; PE, phosphoethanolamine; PG, phosphatidylglycerol; r.t., retention time; SM, sphingomyelin; TG, triglyceride.

Table S6. PLS-DA VIP scores (>1.5) from serum EV-isolated lipids from GD1 patients and controls.

Lipid Species	*m/z*-r.t.	VIP Score
**Positive-ion mode**		
PE(18:0_22:6)	792.5537-7.97	2.3547
SM(d18:1/17:0)	717.5915-7.62	2.333
SM(d18:2/16:0)	701.5595-6.81	2.1367
Cholesterol	369.3515-14.51	2.1141
PE(18:0_20:5)	766.5379-7.75	2.087
PC(18:0_20:5)	808.5842-7.64	2.0593
Plasmenyl-PE(P-16:0/20:4)	724.5271-7.81	2.0407
TG(16:0_18:2_18:2)	872.7705-13.90	1.9468
TG(14:0_18:2_22:6)	892.7392-12.71	1.904
TG(15:0_18:1_18:2)	860.7702-14.14	1.8973
SM(d18:1/18:0)	731.6064-7.95	1.8926
PC(18:0_20:1)	816.6457-9.31	1.8658
Cer-NS(d18:1/24:1)	648.6291-10.14	1.8604
SM(d18:1/16:0)	703.5749-7.32	1.8522
TG(18:1_18:1_22:3)	954.8469-14.94	1.8075
Plasmenyl-PE(P-18:0/18:2)	728.5590-8.59	1.7734
TG(16:0_17:1_20:4)	884.7708-13.94	1.7413
PE(18:0_22:5)	794.5698-8.18	1.7103
TG(14:0_18:3_20:4)	866.7237-12.58	1.7013
SM(d16:1/16:0)	675.5443-6.60	1.697
TG(16:0_22:6_22:6)	968.7695-12.97	1.6758
SM(d16:1/22:0)	759.6377-8.64	1.6731
Plasmenyl-PE(P-18:1/18:2)	726.5414-7.95	1.6676
SM(d18:1/18:1)	729.5901-7.39	1.6405
TG(16:0_18:1_22:6)	922.7848-13.97	1.6357
TG(16:1_18:2_22:6)	918.7543-12.94	1.6217
Plasmenyl-PC(P-16:0/16:0)	718.5746-8.25	1.6182
TG(18:0_18:1_22:4)	954.8490-15.07	1.6068
PC(18:0_22:6)	834.5992-7.85	1.5918
PC(20:2_20:4)	834.5991-7.50	1.5803
Plasmanyl-PC(O-18:0/20:3)	798.6367-8.67	1.577
TG(18:0_18:1_22:5)	952.8315-14.62	1.5727
SM(d18:2/20:0)	757.6229-8.00	1.5583
TG(16:0_16:0_22:6)	896.7700-13.99	1.5543
TG(16:0_18:2_20:5)	894.7548-13.30	1.5405
TG(18:1_20:1_22:4)	980.8638-14.94	1.5378
DG(16:0_18:1)	612.5564-9.13	1.5231
**Negative-ion mode**		
PC(16:0_20:4)	826.5612-7.44	2.3969
PC(16:0_22:5)	852.5763-7.63	2.2157
Plasmanyl-PC(O-16:1/16:0)	762.5676-8.27	2.0609

PC(18:0_22:6)	878.5925-7.86	1.9919
PE(18:0_22:6)	790.5401-8.00	1.9359
Plasmanyl-PC(O-16:0/20:4)	812.5818-7.78	1.9084
HexCer-NS(d18:1/16:0)	744.5632-7.52	1.8689
Plasmenyl-PE(P-16:0/20:4)	722.5140-7.84	1.8465
MSDIAL_ST	465.3047-4.42	1.7927
PE(16:0_22:6)	762.5085-7.41	1.7417
PE(18:0_20:5)	764.5245-7.78	1.6581
Plasmenyl-PE(P-16:0/22:6)	746.5138-7.69	1.6428
HexCer-NS(d18:1/22:0)	828.6587-9.63	1.5902
Plasmenyl-PE(P-18:1/22:6)	772.5300-7.68	1.5389
HexCer-NS(d18:1/24:1)	854.6746-9.55	1.5254
PC(18:0_18:2)	830.5934-8.14	1.523

Cer-NS, ceramide non-hydroxy-fatty acid sphingosine; CTRL, control; DG, diacylglycerol; GD1, Gaucher disease type 1; HexCer-NS, hexosylceramide non-hydroxy fatty acid-sphingosine; PC, phosphatidylcholine; PE, phosphoethanolamine; PLS-DA, Partial Least-Squares Discriminant Analysis; r.t., retention time; SM, sphingomyelin; TG, triglyceride; VIP, Variable Importance in Projection.

Table S7. PLS-DA VIP scores (>1.5) from urine EV-isolated lipids from GD1 patients.

Lipid Species	*m/z*-r.t.	VIP Score
**Positive-ion mode**		
TG(16:0_18:1_22:0)	934.8796-15.82	1.9705
TG(12:0_14:0_16:0)	740.6760-13.04	1.8857
TG(16:0_16:0_20:4)	872.7707-14.20	1.6968
TG(16:0_18:1_18:1)	876.8018-14.75	1.6959
TG(17:0_18:0_18:1)	892.8326-15.31	1.6936
TG(16:0_18:1_24:0)	962.9088-16.09	1.6622
TG(18:0_18:1_20:4)	926.8162-14.70	1.6541
Cer-NS(d18:1/23:0)	636.6295-10.72	1.6433
TG(14:0_16:0_18:2)	820.7386-13.84	1.63
TG(16:0_18:2_18:2)	872.7705-13.90	1.6299
TG(16:0_18:0_18:1)	878.8159-15.16	1.6286
TG(18:1_19:0_20:3)	942.8486-15.08	1.6251
TG(16:0_16:0_18:1)	850.7864-14.77	1.579
PC(18:0_20:2)	814.6318-8.69	1.5683
TG(15:0_15:0_18:1(d7))	829.7984-14.26	1.5461
SM(d17:1/16:0)	689.5597-6.97	1.5407
Cer-NS(d18:1/22:0)	622.6132-10.22	1.5172
TG(16:0_17:0_18:1)	864.8016-14.95	1.5057
**Negative-ion mode**		
LPC(15:0)	526.3153-0.91	2.3941
HexCer-NS(d18:1/16:0)	744.5632-7.52	2.0987
LPE(18:0)	480.3100-2.26	1.938
PC(19:0_19:0)	862.6564-10.15	1.9022
PE(17:0_17:0)	718.5406-8.83	1.8384
Cer-NS(d18:1/16:0)	582.5107-7.99	1.7634
Plasmenyl-PE(P-16:0/20:3)	724.5293-8.11	1.7342
PC(16:0_20:3)	828.5760-7.62	1.5918

Cer-NS, ceramide non-hydroxy fatty acid sphingosine; CTRL, control; GD1, Gaucher disease type 1; HexCer-NS, hexosylceramide non-hydroxy fatty acid sphingosine; LPC, lysophosphatidylcholine; LPE, Lysophosphatidylethanolamine; PC, phosphatidylcholine; PE, phosphoethanolamine; PLS-DA, Partial Least-Squares Discriminant Analysis; r.t., retention time; SM, sphingomyelin; TG, triglyceride; VIP, Variable Importance in Projection.

Table S8. PLS-DA VIP scores (>1.5) from serum EV-isolated lipids from GD1 patients, treatment-naive and post-ERT.

Lipid Species	*m/z*-r.t.	VIP Score
**Positive-ion mode**		
PE(18:0_22:5)	794.5698-8.18	1.8168
OxTG(18:1_18:1_18:2(Ke_or_Epoxy))	914.7788-13.47	1.815
PE(18:0_20:3)	770.5704-8.43	1.7584
TG(18:0_18:0_18:0)	908.8641-15.97	1.7254
PC(19:0_19:0)	818.6633-10.10	1.7084
TG(18:0_18:1_22:4)	954.8490-15.07	1.7054
TG(18:1_18:1_22:3)	954.8469-14.94	1.7052
TG(16:0_18:0_18:1)	878.8159-15.16	1.695
DG(16:0_18:0)	614.5724-9.88	1.6929
TG(14:0_16:0_18:1)	822.7539-14.29	1.6779
PG(16:0_18:2)	764.5447-7.33	1.6694
DG(18:0_18:0)	642.6035-10.85	1.658
TG(14:0_16:0_18:2)	820.7386-13.84	1.6435
TG(15:0_16:0_18:3)	832.7385-13.60	1.5824
TG(16:0_17:1_18:1)	862.7861-14.54	1.5818
TG(18:1_20:1_22:4)	980.8638-14.94	1.5622
TG(16:0_17:0_18:1)	864.8016-14.95	1.5424
TG(18:0_18:1_18:1)	904.8330-15.15	1.5398
TG(14:0_16:0_18:3)	818.7226-13.54	1.5265
TG(14:0_14:0_18:1)	794.7232-13.76	1.5233
TG(17:0_18:1_18:1)	890.8174-14.95	1.5189
TG(18:0_18:1_22:5)	952.8315-14.62	1.5142
**Negative-ion mode**		
PE(18:0_22:6)	790.5401-8.00	2.0688
PE(18:0_20:5)	764.5245-7.78	1.995
PE(18:0_20:3)	768.5561-8.46	1.9879
PE(18:0_22:4)	794.5716-8.64	1.9836
PE(18:0_22:5)	792.5557-8.42	1.9366
PC(16:0_16:1)	776.5465-7.43	1.8807
PE(18:0_20:4)	766.5403-8.19	1.8759
PI(18:0_20:4)	885.5503-7.60	1.8692
Cer-NS(d18:1/24:1)	692.6207-10.19	1.7915
PE(16:0_22:4)	766.5403-8.00	1.7514
PE(16:0_22:6)	762.5085-7.41	1.7121
PC(16:0_18:1)	804.5774-8.02	1.6704
PE(16:0_18:1)	716.5248-8.18	1.6653
Plasmenyl-PE(P-18:0/18:2)	726.5454-8.63	1.6613
PE(16:0_20:4)	738.5084-7.58	1.6333
Plasmanyl-PC(O-18:1/18:2)	814.5982-8.41	1.5239

DG, diacylglycerol; Cer-NS, ceramide non-hydroxy-fatty acid sphingosine; ERT, enzyme replacement therapy; OxTG, oxidized triglyceride; PC, phosphatidylcholine; PE,

phosphoethanolamine; PI, phosphatidylinositol; PG, phosphatidylglycerol; PLS-DA, Partial Least-Squares Discriminant Analysis; r.t., retention time; SM, sphingomyelin; TG, triglyceride; VIP, Variable Importance in Projection.

Table S9. PLS-DA VIP scores (>1.5) from urine EV-isolated lipids from GD1 patients, treatment-naive and post-ERT.

Lipid Species	*m/z*-r.t.	VIP Score
**Positive-ion mode**		
PC(18:0_22:6)	834.5992-7.85	2.3122
TG(18:1_18:2_22:0)	958.8796-15.55	2.2937
TG(16:0_18:1_24:1)	960.8954-15.83	2.2377
TG(18:1_18:2_21:0)	944.8637-15.33	2.0291
DG(18:0_18:1)	640.5877-9.92	2.0073
TG(10:0_16:0_18:2)	764.6746-12.31	1.9763
PE(18:1_20:3)	768.5542-7.84	1.9589
DG(18:1_18:2)	636.5568-8.82	1.9349
PC(18:0_20:3)	812.6155-8.27	1.8753
TG(14:0_16:0_18:3)	818.7226-13.54	1.7484
DG(16:0_18:1)	612.5564-9.13	1.7444
TG(17:0_18:1_20:3)	914.8162-14.62	1.6626
PC(15:0_18:2)	744.5536-7.23	1.5937
TG(15:0_18:1_18:3)	858.7544-13.90	1.5843
TG(15:0_18:2_18:3)	856.7390-13.14	1.5636
PC(15:0_20:4)	768.5536-7.13	1.5617
PC(16:0_18:0)	762.6000-8.60	1.5612
TG(12:0_16:0_18:3)	790.6915-12.55	1.5387
DG(18:0_18:0)	642.6035-10.85	1.5301
TG(18:1_18:1_21:0)	946.8797-15.67	1.5264
**Negative-ion mode**		
Plasmenyl-PE(P-16:0/22:5)	748.5299-8.01	2.768
Plasmenyl-PE(P-18:0/18:2)	726.5454-8.63	2.6065
LPC(16:0)	540.3309-1.19	2.0231
PE(18:1_22:6)	788.5239-7.45	1.6906
HexCer-AP(t18:0/22:0)	862.6628-9.16	1.6684

DG, diacylglycerol; ERT, enzyme replacement therapy; HexCer-AP, alpha-hydroxy hexosylceramide; LPC, lysophosphatidylcholine; PA, phosphatidic acid; PC, phosphatidylcholine; PE, phosphoethanolamine; PLS-DA, Partial Least-Squares Discriminant Analysis; r.t., retention time; SM, sphingomyelin; TG, triglycerides; VIP, Variable Importance in Projection.
